# Effects of Major Families of Modulators on Performances and Gastrointestinal Microbiota of Poultry, Pigs and Ruminants: A Systematic Approach

**DOI:** 10.3390/microorganisms11061464

**Published:** 2023-05-31

**Authors:** Cyrielle Payen, Annaëlle Kerouanton, Jorge Novoa, Florencio Pazos, Carlos Benito, Martine Denis, Muriel Guyard, F. Javier Moreno, Marianne Chemaly

**Affiliations:** 1 French Agency for Food, Environmental and Occupational Health and Safety, ANSES, Hygiene and Quality of Poultry, Pig Products Unit, 22440 Ploufragan, France; 2 Computational Systems Biology Group, National Centre for Biotechnology (CNB-CSIC), Darwin 3, 28049 Madrid, Spain; 3 Instituto de Gestión de la Innovación y del Conocimiento, INGENIO (CSIC and U. Politécnica de Valencia), Edificio 8E, Cam. de Vera, 46022 Valencia, Spain; cbenito@ingenio.upv.es; 4 Instituto de Investigación en Ciencias de la Alimentación (CIAL), CSIC-UAM, CEI (UAM + CSIC), Nicolás Cabrera 9, 28049 Madrid, Spain

**Keywords:** microbiota, performance, poultry, pigs, ruminants

## Abstract

Considering the ban on the use of antibiotics as growth stimulators in the livestock industry, the use of microbiota modulators appears to be an alternative solution to improve animal performance. This review aims to describe the effect of different families of modulators on the gastrointestinal microbiota of poultry, pigs and ruminants and their consequences on host physiology. To this end, 65, 32 and 4 controlled trials or systematic reviews were selected from PubMed for poultry, pigs and ruminants, respectively. Microorganisms and their derivatives were the most studied modulator family in poultry, while in pigs, the micronutrient family was the most investigated. With only four controlled trials selected for ruminants, it was difficult to conclude on the modulators of interest for this species. For some modulators, most studies showed a beneficial effect on both the phenotype and the microbiota. This was the case for probiotics and plants in poultry and minerals and probiotics in pigs. These modulators seem to be a good way for improving animal performance.

## 1. Introduction

The aim of the livestock industry is to reach high productivity and good quality at the lowest possible cost. This is why poultry, pigs and ruminants are genetically selected to gain the maximum weight in the minimum possible time with the minimum required feed [1,2,3]. This phenotype is mainly characterised by the Feed Conversion Ratio (FCR), representing the efficiency with which the animal converts feed into increased body mass. A low FCR indicates that animals gain considerable weight from small amounts of feed. Increasing evidence appears to show that the Gastrointestinal Tract (GIT) microbiota may play a central role in the acquisition of this phenotype [4,5,6]. In fact, it has been found that bacterial diversity within the GIT is higher in poultry with lower FCR values [7]. A high abundance of certain bacterial genera, such as *Lactobacillus* or *Ruminococcus*, has been found to promote performance in chicks [8,9,10], while an abundance of genera such as *Prevotella*, *Akkermansia* and *Campylobacter* is negatively correlated with weight gain or feed efficiency [8,10,11]. Similarly, for pigs, feed efficiency is correlated with the composition of the gastrointestinal microbiota. For example, an abundance of *Lactobacillus* and *Ruminococcus* is positively correlated with better feed efficiency [12,13,14], while pigs with a high FCR have a greater abundance of *Prevotella* and *Campylobacter* [12,13,14]. In ruminants, *Ruminococcus* is also associated with a greater Average Daily Gain (ADG), and *Prevotella* is associated with a lower ADG [15]. Considering these data and the ban on the use of antibiotics as growth promoters, other microbiota modulators are increasingly being used as a solution to improve animal performance. In the same way, some microbiota modulators can have negative effects on the phenotype by modulating GIT microbiota. Therefore, this present work, which is part of the European project RIMICIA, aims to describe the impact of different families of modulators on the GIT microbiota of poultry, pigs and ruminants, including antibiotics as negative control and the potential consequences on the host physiology.

## 2. Materials and Methods

This systematic review was carried out following the Preferred Reporting Items for Systematic Review and Meta-Analysis (PRISMA) protocols [16]. The research question to be reviewed was: What are the impacts of the main feed modulators on the intestinal microbiota composition and performances of poultry, pigs and ruminants?

### 2.1. Literature Search

This review focused on controlled trials and systematic reviews that evaluated the effects of modulators on the GIT microbiota in poultry, pigs and ruminants. PubMed identifies controlled trials and systematic reviews, but this is not the case with Scopus and Web of Science. As a result, only PubMed could be used for this work. The search was based on Medical Subject Headings (MeSH) in titles, abstracts and keywords. The following search criteria were designed by two researchers and used:

For poultry: (((“gut microbiota”) OR (“intestinal microbiota”) OR (“gastrointestinal microbiota”) OR (“gut microbiom”) OR (“intestinal microbiom”) OR (“gastrointestinal microbiom”) OR (“gut microflora”) OR (“intestinal microflora”) OR (“gastrointestinal microflora”)) AND ((poultry) OR (chicken) OR (turkey) OR (duck) OR (geese) OR (“gallus gallus”) OR (hen) OR (bird*) OR (fowl*) OR (broiler))).

For pigs: (((“gut microbiota”) OR (“intestinal microbiota”) OR (“gastrointestinal microbiota”) OR (“gut microbiom”) OR (“intestinal microbiom”) OR (“gastrointestinal microbiom”) OR (“gut microflora”) OR (“intestinal microflora”) OR (“gastrointestinal microflora”)) AND ((pig*) OR (swine) OR (suidae) OR (sus scrofa) OR (minipig))).

For ruminants: (((“gut microbiota”) OR (“intestinal microbiota”) OR (“gastrointestinal microbiota”) OR (“gut microbiom”) OR (“intestinal microbiom”) OR (“gastrointestinal microbiom”) OR (“gut microflora”) OR (“intestinal microflora”) OR (“gastrointestinal microflora”)) AND ((cattle) OR (livestock) OR (bovine) OR (bovins) OR (sheep) OR (goat))).

### 2.2. Inclusion and Exclusion Criteria

Five parameters, named PICOS for Population, Intervention, Comparison, Outcomes and Study design, were considered to determine the inclusion and exclusion criteria (Table 1). No restriction on the year of publication was used. At the first round, all studies that were not controlled trials or systematic reviews, as well as duplicate studies, were excluded. Then, using the PICOS table criteria, articles were included or excluded based on the keywords in the title and abstract. All these steps were performed using the PMIDigest system [17]. For each animal species, all the articles resulting from the PubMed searches above were used as input for PMIDigest, which generates an interactive web report to facilitate the manipulation and extraction of relevant information from the sets of articles. These interactive web reports also allow identification of controlled trials and systematic reviews and quickly exclude articles matching the exclusion criteria based on the keywords found in the title or abstract.

The web reports for the three species are available at:

https://csbg.cnb.csic.es/RIMICIA/Poultry_p.html (accessed on 2 May 2023);
https://csbg.cnb.csic.es/RIMICIA/Pigs_p.html (accessed on 2 May 2023);
https://csbg.cnb.csic.es/RIMICIA/Ruminants_p.html (accessed on 2 May 2023).

At the last step, articles were included or excluded based on full reading and use of the PICOS table. These steps were performed by one researcher.

### 2.3. Data Extraction

Controlled trials were classified according to families of modulators that were previously determined by the consortium of the RIMICIA project: 1. Macronutrients including prebiotics, fibres, enzymes, lipids and amino acids; 2. Micronutrients including minerals, polyphenols, vitamins and immunoglobulin; 3. Microorganisms and derivatives including probiotics, zoonotic/pathogenic bacteria, symbiotic and postbiotics; 4. Antimicrobial agents including antibiotics and antimicrobial peptides; and 5. Plants, seaweeds and derived products. If a controlled trial considered several families of modulators, it was included in each of these families. In the discussion, for each animal species, the modulators are described from the family with the greatest number of articles to the family with the least.

For each included controlled trial, relevant information related to authors, publication year, experimental conditions (species/strain, gender and health), methods (dose, time of intervention and microbiota characterisation) was extracted. Similarly, the extracted results made it possible to answer questions about: phenotype of the host (weight, food intake, FCR, villi structure, immunity, intestinal barrier) and microbiota (diversity, phylum abundance, lactic-acid-producing bacteria abundance, short-chain fatty acids (SCFA)-producing bacteria abundance, pH, SCFA concentration and pathogenic and/or zoonotic bacteria abundance). Based on these parameters, it was determined whether the modulators had a positive, negative or no effect on the microbiota and on the host phenotype compared to the control group (without modulator), which represented the “normal” microbiota for the study conditions for each study. These data are listed in the tables presented as Supplementary Data.

## 3. Results

### 3.1. Selected Studies

**Figure 1:**
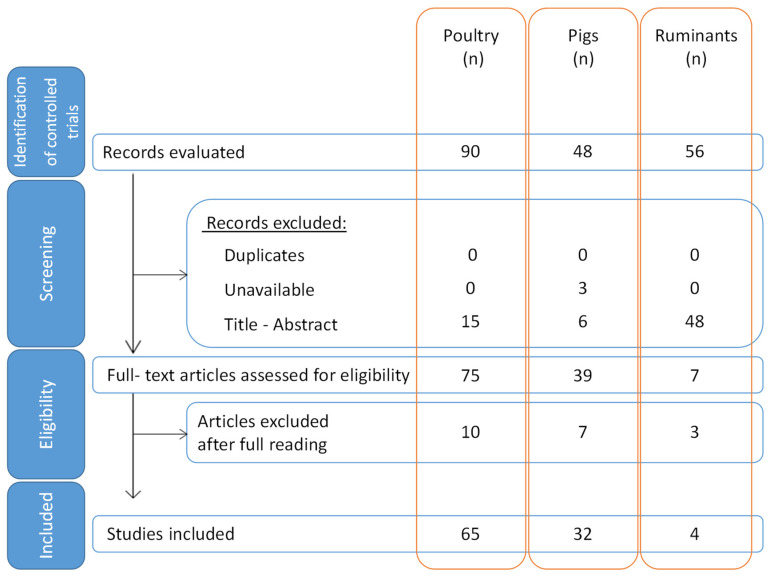
Flow diagram of the literature search process with the number of papers at each step of the process for poultry, pigs and ruminants.

Our literature search flow diagram (Figure 1) shows that 90 controlled trials or systematic reviews were identified in PubMed for poultry, 48 for pigs and 56 for ruminants. Of these, no duplicates were identified and excluded. Three articles were unavailable and were therefore excluded. Using the PICOS table (Table 1) and based on titles and abstracts, 15 articles were excluded for poultry and 6 for pigs. For ruminants, 48 controlled trials were excluded; most of these articles focused on modulation of the human intestinal microbiota in the case of intolerance or allergy to cow’s milk. The remaining 75 articles for poultry, 39 for pigs and 7 for ruminants were retained, but, after full reading, 10, 7 and 3 articles were excluded, respectively, based on the PICOS table. Finally, for this review, 65 articles were eligible for poultry, 32 for pigs and 4 for ruminants. All included studies for poultry were published from 2004 to 2021. For pigs, the assessed studies were published from 2006 to 2021. Finally, studies on ruminants were published between 2018 and 2021.

### 3.2. Distribution of Controlled Trials According to Modulator Family

**Figure 2:**
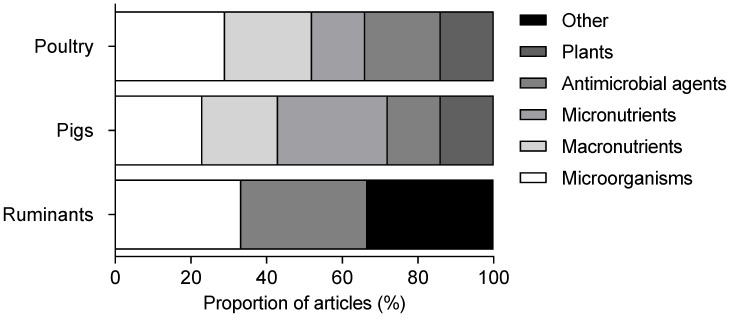
Distribution of controlled trials according to modulator families in poultry, pigs and ruminants.

In this selection of articles, microorganisms and derivatives were the most commonly studied family of modulators in poultry, with 29% of articles focusing on them. Within this family, probiotics and zoonotic and/or pathogenic bacteria were the most represented. They were followed by the macronutrients family, mainly prebiotics, with 23% of articles. Antimicrobial agents represented 20% of articles, primarily antibiotics (Figure 2).

In pigs, the micronutrients family was the most commonly studied, with 29% of controlled trials. Minerals were the most represented group in this family. Microorganisms and derivatives accounted for 23% of the articles on pigs, the majority related to probiotics. Macronutrients were also studied in 20% of the articles, principally prebiotics (Figure 2).

With only four articles selected for ruminants, it was difficult to conclude on the modulators of interest for this species. These articles dealt with microorganisms, micronutrients and other families (Figure 2).

### 3.3. Effect of Modulators on Intestinal Microbiota and Phenotype of Poultry

#### 3.3.1. Microorganisms and Derivatives

##### Probiotics

Probiotics are exogenous and non-pathogenic microorganisms introduced into the intestinal flora to modulate it in order to improve microbial balance in the GIT. In the selected articles, the most frequently studied probiotics in poultry were related to the bacterial genus *Bacillus* [18,19,20,21,22,23]. In the majority of cases, probiotics improved poultry performance with an increase in body weight [18,19,20,24,25] associated with a decrease in the FCR [18,20,24]. Probiotics also had a beneficial effect by reducing oxidative stress [19,24], increasing lymphocyte concentrations and the production of antibodies [25,26] and modulating cytokine concentrations [23]. Concerning the intestinal microbiota, in the majority of studies, probiotics induced an increase in the abundance of lactic-acid-producing bacteria, in particular, those of the genus *Lactobacillus* and *Bifidobacterium* [19,20,21,23,24,25]. The effect of probiotics on the abundance of SCFA-producing bacteria was inconsistent, for example, supplementation with *Bacillus amyloliquefaciens* increased the abundance of *Faecalibacterium* in the caecum, while supplementation with *Bacillus subtilis* decreased its abundance in the small intestine [18,21]. Probiotics were able to reduce the abundance of zoonotic and/or pathogenic bacteria such as *Escherichia coli*, *Salmonella* and *Clostridium perfringens* [19,20,22,25,27,28]. All these results demonstrated an effect of probiotics on the intestinal microbiota by increasing the abundance of lactic-acid-producing bacteria and decreasing the abundance of zoonotic and/or pathogenic bacteria. This change in the microbiota composition was associated with improvement in performance and the immunity status of poultry (Supplementary Data S1—Table S1).

##### Zoonotic and/or Pathogenic Bacteria

In the selected articles, *Clostridium perfringens*, *Escherichia coli* and *Salmonella* were the most frequently studied zoonotic and/or pathogenic bacteria in poultry [29,30,31,32,33,34,35]. These studies demonstrated the deleterious effect of these bacteria on poultry performance, which was characterised by decreased body weight [29,31,32,33] and increased FCR [29,35]. These performance changes were associated in many cases with a decrease in the size of the villi in the ileum and jejunum [29,32,33], reflecting a decrease in the absorption surface at the level of the small intestine. Infections by these bacteria were also associated with stimulation of the immune system, which mainly resulted in increased concentrations of pro-inflammatory makers such as TNFα [28,29,33]. In addition, these pathogenic bacteria disrupted the integrity of the intestinal barrier by negative modulation of tight junctions and increased permeability [28,29]. Regarding the microbiota, these pathogenic and/or zoonotic bacteria in most cases decreased the abundance of lactic-acid-producing bacteria such as *Lactobacillus* [29,31,32,33,34]. This was associated with an increased abundance of certain pathogenic bacteria other than those administered to the animals studied. For example, the administration of *Salmonella pullorum* to chickens led to an increase in the abundance of *Escherichia coli* [28,33]. Zoonotic and/or pathogenic bacteria are therefore capable of inducing profound disturbances of the host phenotype, which result in reduced performance linked to a reduction in the intestinal absorption surface and an alteration of the intestinal barrier associated with an increase in pro-inflammatory markers. These bacteria are also able to profoundly disrupt the composition of the intestinal microbiota by decreasing the abundance of lactic-acid-producing bacteria and increasing the abundance of zoonotic and/or pathogenic bacteria (Supplementary Data S1—Table S2).

##### Probiotics Combined with Another Family of Modulators

In some of the studies, probiotics were combined with the administration of organic acids or fermented products. Unlike using probiotics alone, these combinations did not induce changes in poultry performance, reflected by the FCR [22,33,36,37]. However, this type of supplementation led to decreased concentrations of inflammatory markers such as TNFα, INFγ, IL-6 or IL-1β in the digestive system [33,36]. Regarding the microbiota, these modulators had variable effects on the abundance of lactic-acid-producing bacteria [22,33,36,37]. However, they appeared to enable a reduction in the abundance of zoonotic and/or pathogenic bacteria such as *Salmonella* or *Escherichia coli* [22,33]. Therefore, when combined with another modulator, probiotics seem to have a weaker effect on the phenotype. The effects on the microbiota are small but interesting since they make it possible in certain conditions to reduce the abundance of zoonotic and/or pathogenic bacteria (Supplementary Data S1—Table S3).

##### Synbiotics

A synbiotic is a combination of one or more probiotics with one or more prebiotics. The use of synbiotics in poultry did not affect poultry performance [38,39], although one of these studies observed an increase in the height of the intestinal villi [39]. However, in most cases, synbiotics increased the abundance of lactic-acid-producing bacteria, such as *Lactobacillus*, *Bifidobacterium* and *Pediococcus*, and reduced the abundance of zoonotic and/or pathogenic bacteria such as *Escherichia coli* and *Salmonella* [38,39]. However, one of the three studies looking at the synbiotics showed no effect on the composition of the gut microbiota [40]. Therefore, according to these three studies, the combination of a prebiotic with a probiotic does not seem to increase the effects induced by the probiotic alone (Supplementary Data S1—Table S4).

##### Postbiotics

In this work, postbiotics are considered to be bioactive compounds produced by probiotic bacteria and that confer a health benefit to the host. In poultry, the most frequently studied postbiotics were lactic acid, propionic acid and butyric acid. Whether postbiotics were used alone or in combination, they did not have any effect on the phenotype or abundance of lactic-acid-producing bacteria such as *Lactobacillus* or *Enterococcus* in poultry [22,41,42,43]. However, some isolated observations suggested that postbiotics could have beneficial effects, in particular by reducing the abundance of *Escherichia coli* [22] and by increasing the concentration of organic acids other than that administered [41]. More studies appear to be needed to confirm these beneficial effects (Supplementary Data S1—Table S5).

#### 3.3.2. Macronutrients

##### Prebiotics

Prebiotics are non-digestible substances (often oligosaccharides or polysaccharides) that selectively promote the growth and/or activity of certain bacteria and that provide a health benefit. Inulin and oligosaccharides were the most frequently studied prebiotics in poultry [30,35,44,45,46,47,48,49]. In most studies, the use of prebiotics in poultry did not affect animal performance (body weight, feed intake or FCR) [44,45,46,47,48]. This was supported by the lack of change in intestinal villus size following prebiotic supplementation [35,45,47]. In the majority of cases, this supplementation induced an increase in the abundance of lactic-acid-producing bacteria such as *Lactobacillus, Bifidobacterium* and/or *Enterococcus* [30,35,44,45,47,48,49]. However, it was not consistently associated with decreased intestinal or caecal pH [35,47,48]. Finally, in some cases, the use of prebiotics reduced the abundance of zoonotic and/or pathogenic bacteria such as *Clostridium perfringens* and *Salmonella enteritidis* [30,35,45]. Although prebiotics did not seem to have an effect on poultry performance, it appears that this type of supplementation is able to induce changes in the composition of the microbiota by promoting an increase in the abundance of lactic-acid-producing bacteria and, in some cases, a decrease in the abundance of zoonotic and/or pathogenic bacteria (Supplementary Data S1—Table S6).

##### Fibres

Fibres are plant food components not transformed by digestion. The fibres tested on poultry induced variable modulations of body weight or feed intake without modulating the FCR [46,50,51], which was explained by the absence of modification of the structure of the villi of the small intestine [46,50,51,52]. In some studies, the fibres were able to induce an increase in the abundance of lactic-acid-producing bacteria and a decrease in the abundance of zoonotic and/or pathogenic bacteria such as *Salmonella* and *Escherichia coli* [30,46,52]. Although fibres were able to induce beneficial effects on the composition of the gastrointestinal microbiota of poultry by increasing lactic-acid-producing bacteria and decreasing zoonotic and/or pathogenic bacteria, the effects on poultry performance were not obvious (Supplementary Data S1—Table S7).

##### Enzymes

In the selection of articles, the enzymes of interest in poultry were phytase or glucanases in combination with xylanases [47,51,53,54,55]. Glucanase combined with xylanase did not induce an improvement in body weight, feed intake or FCR, which was partly explained by the absence of modulation of the structure of the intestinal villi [47,51,54]. However, phytase induced an increase in body weight gain [53,55]. Most studies showed that enzymes had no effect on the abundance of lactic-acid-producing bacteria or even on intestinal and caecal pH [47,51,54,55]. In addition, the enzymes failed to modulate the abundance of zoonotic and/or pathogenic bacteria [51,53,54]. It therefore appears that the use of enzymes in poultry does not induce a significant effect either on the phenotype or on the microbiota (Supplementary Data S1—Table S8).

##### Enzyme and Fibres

Based on only two articles dealing with the combination of enzymes and fibres, no impact on the microbiota was found. The addition of fibres to the enzymes did not induce any effect on the abundance of lactic-acid-producing bacteria, the pH, the concentration of SCFAs or the abundance of zoonotic and/or pathogenic bacteria [47,51] (Supplementary Data S1—Table S9).

##### Lipids

Of the three studies focusing on the effect of lipids on poultry, only one looked at the effect on phenotype and immunity [56]. This study showed that the administration of lauric acid induced weight gain, associated with an increase in the size of the villi in both the ileum and duodenum. Moreover, this study showed an increase in the production of antibodies and a decrease in pro-inflammatory cytokine concentrations [56]. Concerning the microbiota, it seems that lipids did not induce changes in caecum α-diversity [34,42,56]. However, the lipids appeared to be able to modify the relative abundance of the phyla, with, in particular, a decrease in the abundance of *Proteobacteria* [34,42]. Although lipids did not appear to modulate the abundance of lactic-acid-producing bacteria, they modulated the abundance of SCFA-producing bacteria, but not in similar ways between studies and intestinal sections (i.e., ileum, jejunum, caecum) [34,42,56]. One of the studies showed an increase in the abundance of *Faecalibacterium* and a decrease in the abundance of *Phascolarctobacterium*, which are two SCFA-producing bacteria and a decrease in concentrations of acetate, butyrate and propionate in the caecum of poultry [56]. Therefore, these studies demonstrated that the use of lipids does not induce changes in α-diversity but leads to a change in phylum abundance (Supplementary Data S1—Table S10).

##### Amino Acids

Only one controlled trial looked at the effect of amino acids in poultry [57]. This study showed that L-arginine had beneficial effects on both phenotype (an increase in body weight and a decrease in the FCR) and microbiota composition (increase in *Firmicutes* abundance and decrease in *Proteobacteria* abundance), suggesting that the use of amino acids could be beneficial for poultry (Supplementary Data S1—Table S11).

#### 3.3.3. Antimicrobial Agents

##### Antibiotics

The most frequently studied antibiotic in poultry was bacitracin. Studies on the effects of antibiotics on poultry showed variable results. Concerning the phenotype, while some studies showed a beneficial effect on the performance of the animals, resulting in increased body weight gain [18,24,25,29,49,56,58,59] and/or decreased FCR [24,29,35,41,58,60], others reported an absence of change in performance [21,35,39,42,43,57]. These results varied within the same family of antibiotics. Similarly, the results on the structure of the villi of the small intestine were variable across studies. Indeed, three studies showed an increase in villi height [23,29,56], while two highlighted a decrease [41,60], and one found an absence of effect [52]. Antibiotics led to an increase in antibody concentrations and a decrease in interleukin concentrations [23,25,40,49,58]. In addition, antibiotics increased the antioxidant capacity [24,57,58,59]. At the level of the microbiota, antibiotics were able to reduce α-diversity [21,61], although, in most cases, no effect was observed [24,42,56,57]. Modulation of the relative abundance of the three main phyla generally found in poultry appeared following administration of antibiotics. This modulation was often in favour of *Proteobacteria* and *Firmicutes* and against *Bacteroidetes* [21,56,58,61]. The effect of antibiotics on the abundance of lactic-acid-producing bacteria was inconsistent. In a few cases, antibiotics reduced the abundance of SCFA-producing bacteria [18,21,29,56,58]. However, no effect on pH or SCFA concentrations was described [35,40]. In the majority of cases, antibiotics induced a decrease in the abundance of zoonotic and/or pathogenic bacteria [29,35,39,56,58,59,61], although an increase in their abundance was observed in a few other cases [25,56,60]. Therefore, although the use of antibiotics induces variable effects on the phenotype and the composition of the intestinal microbiota of poultry, it appears that they are able to improve the performance of the animals but also to profoundly modify the composition of the intestinal microbiota by acting mainly on the abundance of phyla and on the abundance of SCFA-producing bacteria (Supplementary Data S1—Table S12).

##### Antimicrobial Peptides

Only one study focusing on the effect of antimicrobial peptides in poultry was found among the selection of controlled trials [29]. This study revealed that the administration of cLF36 led to beneficial effects on the phenotype, with a decrease in the FCR and an improvement in the intestinal barrier. In the microbiota, cLF36 caused an increase in the abundance of *Lactobacillus* and a decrease in the abundance of *Escherichia coli* [29]. These data suggest that the use of antimicrobial peptides in poultry might have beneficial effects on both the phenotype and the composition of the gut microbiota (Supplementary Data S1—Table S13).

#### 3.3.4. Plants and Seaweed

Many of the selected articles focused on the effect of various plants and seaweed on the phenotype and gut microbiota of poultry. These supplementations either had no effect on the phenotype [26,62,63,64] or induced an increase in performance, resulting in increased body weight associated in some cases with reduced FCR and an increase in the size of the intestinal villi [45,65,66,67]. The plants modified the immune system status of poultry by increasing antibody and antioxidant enzyme concentrations [57,65,67,68]. Regarding the microbiota, plants and seaweed led to an increase in the abundance of *Lactobacillus* in the majority of cases [26,31,45,57,58,60,62,65,66,67]. Plants and seaweed did not induce changes in SCFA-producing bacteria [62,63]. This type of supplementation either had no effect [26,65] or induced a reduction in the abundance of zoonotic and/or pathogenic bacteria [45,58,64,66,67,68,69]. The use of plants in poultry therefore involves beneficial effects on both phenotype and microbiota (Supplementary Data S1—Table S14).

#### 3.3.5. Micronutrients

##### Minerals

Zinc and iron were the two most frequently studied minerals in poultry. Although the minerals appeared to induce, in certain cases, an increase in body weight [32,44,70,71] and in the size of the intestinal villi [32,71,72], they did not improve performance, which resulted in reduced FCR [32,53,70,72,73]. The consequences of mineral supplementations on α- or β-diversity were highly variable [32,72,74,75,76,77]. Despite this, it was observed that minerals could induce profound changes in the composition of the gut microbiota by modulating the abundance of phyla [72,75,76], lactic-acid-producing bacteria [32,44,53,70,72,74,75,76], SCFA-producing bacteria [53,72,74,76,77] and zoonotic and/or pathogenic bacteria [44,70,71,72,76]. However, the way in which minerals regulated the abundance of these different bacteria was highly variable from one study to another. Therefore, mineral supplementation in poultry does not seem to induce a beneficial effect on animal phenotype, despite profound modulation of the gastrointestinal microbiota (Supplementary Data S1—Table S15).

##### Polyphenols

Only one study in our article selection was on the effect of polyphenols administered alone in poultry. This study showed that chestnut tannins led to an increase in body weight, as well as an increase in villi size, without modulating the FCR of poultry. This supplementation decreased the concentration of the pro-inflammatory interleukin Il-6 and increased the concentration of antioxidant enzymes such as SOD and TAOC, leading to a decrease in the total oxidant status. Moreover, this polyphenol increased ZO-1 expression, a tight junction protein that helps maintain intestinal permeability. At the microbiota level, tannins induced a decrease in the abundance of *Escherichia coli* without modulating the abundance of *Lactobacillus* in both the caecum and ileum [78]. This study suggests that polyphenols could benefit both gut microbiota and poultry phenotype (Supplementary Data S1—Table S16).

##### Vitamins

Similarly, only one study was on the effect of vitamins on the phenotype and the intestinal microbiota of poultry. In this study, vitamin E did not induce modulation of the phenotype (no changes in body weight or FCR). This supplementation led to an increase in the abundance of *Escherichia coli* in the ileum but not in the caecum. Moreover, no changes in lactic-acid-producing bacteria abundance were observed [64] (Supplementary Data S1—Table S17). This study therefore suggests that vitamins could have deleterious effects on the intestinal microbiota in poultry. However, as for polyphenols, additional data are needed to better define the effects of these modulators on poultry phenotype and microbiota.

### 3.4. Effect of Modulators on Intestinal Microbiota and Phenotype of Pigs

#### 3.4.1. Micronutrients

##### Minerals

Minerals, specifically zinc and copper, represent the most frequently investigated modulator in the selection of articles concerning pigs. Although some studies showed an absence of effect on pig performance [79,80,81], the administration of minerals increased ADG [80,82,83,84], which could in some cases be associated with a decrease in the FCR [83,84]. This result could be due to the decreased rates of diarrhoea observed in pigs supplemented with minerals [81,83,84]. A study highlighted the increase in the size of jejunal villi following copper supplementation, which could also explain the increase in pig performance [80]. A few changes in immune response were described; however, this type of supplementation seemed to induce an increase in the expression of antioxidant enzymes such as GPx, Atox 1 and SOD1 [80,84]. Concerning the intestinal barrier, the results highlighted an absence of effect on intestinal permeability [82,83]. Regarding the microbiota, most of the selected studies showed no change in α-diversity [82,83]. A beneficial effect on the microbiota was observed with, in some cases, an increase in the abundance of lactic-acid-producing bacteria, such as *Lactobacillus* and *Lactococcus* [80,83,85], and a decrease in the abundance of *Escherichia coli* [80,82]. However, a decrease in the abundance of SCFA-producing bacteria such as *Roseburia* was also observed in response to mineral administration, but no data were available to determine the consequences on SCFA concentrations [81,85]. The minerals were therefore able to induce an improvement in pig performance. This seemed to be associated with changes in the microbiota mainly characterised by an increase in the abundance of lactic-acid-producing bacteria (Supplementary Data S2—Table S18).

##### Polyphenols

The assessment of relevant studies addressing the effect of polyphenols on the phenotype and the composition of the microbiota in pigs yielded only a few results. One of the two studies examined the effect of tannins and showed improved performance with an increase in average body weight and a decrease in the FCR. It was not associated with a change in villi height in the jejunum and caecum. This study did not demonstrate changes in the composition and metabolism of the microbiota [86]. The second study did not investigate the impact of polyphenols on pig performance, but it did show a decrease in the abundance of the *Acidobacteria* phylum, associated with a decrease in the abundance of *Enterococcus* lactic-acid-producing bacteria [87]. Therefore, additional data are needed to better define the effects of polyphenols on porcine phenotype and microbiota composition (Supplementary Data S2—Table S19).

##### Immunoglobulin

Only one of the selected studies examined the consequences of immunoglobulin supplementation on pig microbiota. It showed that immunoglobulin Y supplementation led to decreased diarrhoea rates, without increasing pig performance. The inflammatory status in the jejunum was not changed. Concerning microbiota composition, despite a decrease in the abundance of *Enterococcus* lactic-acid-producing bacteria, immunoglobulin Y induced a decrease in the abundance of *Escherichia coli* [88]. Therefore, although only one study was identified for immunoglobulins, the effect on diarrhoea and the abundance of *Escherichia coli* make it a supplementation of interest for pigs (Supplementary Data S2—Table S20).

#### 3.4.2. Microorganisms and Derivatives

##### Probiotics

Concerning pigs, most selected articles focused on the effect of multistrain probiotics. The administration of probiotics in pigs consistently induced improved performance, which was reflected in increased body weight and/or average ADG [84,89,90,91], decreased FCR [84,90,91,92] and decreased diarrhoea rates [84,89,92,93]. These results were associated with an increase in the height of jejunal villi [92,93,94] and digestibility capacity [90,91,94]. In addition, probiotics did not seem to modify the immune status of animals [79,84,90,94]. The results of microbiota diversity were variable, although probiotics appeared to profoundly modulate the microbiota of pigs [89,93,95]. Interestingly, the relative abundance of phyla was often modulated in favour of *Firmicutes* [82,95]. Probiotics either had no effect on the abundance of lactic-acid-producing bacteria [79,89,91,94,95] or had a beneficial effect by increasing their abundance [82,84,90,92]. In contrast, the effect on SFCA-producing bacteria was less evident [89,93]. Finally, probiotics induced a decrease in the abundance of the genus *Escherichia* in the majority of cases [82,84,90,94]. In pigs, probiotics were therefore able to induce performance improvements. This could be observed in parallel with changes in the composition of the microbiota, such as an increase in the abundance of lactic-acid-producing bacteria, which could also explain the decreased abundance of the genus *Escherichia* (Supplementary Data S2—Table S21).

##### Probiotics Combined with Other Modulators

When probiotics were combined with other modulators, such as minerals or vitamins, the results were similar to those obtained when using probiotics alone: improvement or no change in performance, characterised by an increase in body weight and a decrease in the FCR and inconsistent modification of lactic-acid-producing bacteria abundance [84,96]. Associated with other modulators, probiotics could also induce a decrease in the abundance of *Escherichia coli* [84]. Therefore, probiotics associated with other modulators have either an absence of effect or beneficial effects on the microbiota and/or the phenotype in pigs (Supplementary Data S2—Table S22).

##### Postbiotics

The use of SCFAs in pigs did not induce any effect on host phenotype, except when combined with caprylic acid, a medium-chain fatty acid. This combination led to an increase in body weight and in crude digestibility, with no change in the FCR [97]. Administration of sodium butyrate or propionic acid + formic acid did not increase their concentrations in the ileum, jejunum and caecum [86,97]. However, an increase in valerate and isobutyrate concentrations was observed after propionic acid + formic acid supplementation [97]. Finally, postbiotics did not seem to reduce the abundance of pathogenic and/or zoonotic bacteria such as *Escherichia coli* and *Clostridium perfringens* [97]. Therefore, additional data are needed to better define the effects of postbiotics on porcine phenotype and microbiota composition (Supplementary Data S2—Table S23).

##### Zoonotic and/or Pathogenic Bacteria

Unlike for poultry, only one article focused on the effect of zoonotic and/or pathogenic bacteria in pigs. This controlled trial studied the combined effect of *Salmonella* typhimurium, *Salmonella* enteritidis, *Salmonella* choleraesuis, *Escherichia coli* and *Clostridium perfringens* on the phenotype and microbiota of pigs. This combination of zoonotic and/or pathogenic bacteria impaired host performance with decreased body weight gain, which was not associated with a change in the FCR. No change in villus structure was reported, but body weight modifications could be explained by the decrease in average daily feed intake. An increase in diarrhoea rates was observed. In addition, this infection increased IgA concentrations and reduced the abundance of lactic-acid-producing bacteria: *Lactobacillus* in the ileum and *Bifidobacterium* in the caecum [92]. These data therefore demonstrated a deleterious effect of bacterial infection on both the phenotype and the microbiota of the pigs (Supplementary Data S2—Table S24).

#### 3.4.3. Macronutrients

##### Prebiotics

The effect of prebiotics on the phenotype of pigs was insufficiently described in our selection of articles, but prebiotics were shown to lead to positive effects on performance, such as an increase in body weight associated with an increase in villi height in both the jejunum and ileum [98] and an increase in feed intake associated with a decrease in diarrhoea rates [81]. The effects of prebiotics on the microbiota demonstrated an absence of effect on the abundance of phyla in the majority of cases [81,98]. The modulation of lactic-acid-producing bacteria abundance was variable; galacto-oligosaccharides induced an increase in *Lactobacillus* abundance in the ileum [98], while inulin did not modify *Lactobacillus* abundance in the colon [99]. Prebiotics did not influence SFCA-producing bacteria abundance [81,98], while total SCFA concentrations [98] and butyrate concentrations [99] were modified. The use of prebiotics in pigs induced highly variable phenotype and microbiota composition responses, highlighting the need for additional data to better characterise their effects (Supplementary Data S2—Table S25).

##### Fibres

Only two controlled trials examined fibre supplementation in pigs. None of these studies determined weight, food intake or the FCR. Fibres were able to increase α-diversity [100], as well as richness [101], and to modify β-diversity [100]. This was associated with increased abundance of the phylum *Bacteroidetes* [100]. However, neither of the two studies showed a change in the abundance of lactic-acid-producing bacteria in the faeces [100,101]. Guar gum + cellulose supplementation resulted in increased abundance of SCFA-producing bacteria *Roseburia* and *Bacteroides*. This increase was associated with increased concentrations of butyrate and propionate [100]. These data suggest a beneficial effect of fibres on the composition of the faecal microbiota of pigs. However, additional data are needed, in particular to characterise their effect on the phenotype of the animals and on the abundance of zoonotic and/or pathogenic bacteria (Supplementary Data S2—Table S26).

##### Amino Acids

One study examined the effect of amino acid administration on animal performance. This study showed that, unlike L-aspartate, D-aspartate administered alone induced a decrease in ADG. However, as with L-aspartate, this did not lead to a change in the FCR [102]. The inflammatory and oxidative state was modified by the administration of amino acids. Additionally, supplementation with *N*-acetyl cysteine resulted in an increase in the expression of the antioxidant enzyme GPx and the total oxidant capacity in both the caecum and colon [103]. N-aspartate and/or L-aspartate supplementation decreased the expression of NOD1, a protein involved in apoptosis, and of TLR4, a membrane receptor involved in innate immune response [102]. The effect on the microbiota was highly variable from one study to another [102,103,104]. The only highlighted fact was a decrease in the abundance of the genus *Escherichia* [102,103]. These studies suggest that amino acids are able to modify the inflammatory and oxidative state in pigs, but more data are needed to characterise their effect on the phenotype and composition of the gastrointestinal microbiota (Supplementary Data S2—Table S27).

##### Proteins

Only one of the selected controlled trials focused on the consequences of variations in protein levels on performance and microbiota composition in pigs. This study showed no modulation of the phenotype (body weight, FCR or diarrhoea rate) [81]. Concerning the microbiota, the increase in the level of proteins led to an increased abundance of *Proteobacteria* in the colon, without modulating α-diversity or the abundance of the SCFA-producing bacteria *Roseburia* and *Bacteroides* or that of the zoonotic bacteria *Campylobacter* [81] (Supplementary Data S2—Table S28).

#### 3.4.4. Antimicrobial Agents

##### Antibiotics

Although the administration of antibiotics in pigs could induce an increase in body weight gain [91,94,105,106], sometimes associated with a decrease in the FCR [91,105], some results described no effect on performance [88,96]. The majority of studies showed no effect on α-diversity [96,105,106]. The two studies focusing on the abundance of phyla showed that antibiotics disrupted the composition of phyla, characterised mainly by an increase in the abundance of *Firmicutes* [95,105]. An increase in the abundance of SCFA-producing bacteria such as *Blautia* and *Phascolarctobacterium* was observed [105]. The effects of antibiotics on the abundance of lactic-acid-producing bacteria were variable [88,91,94,95,96,106]; however, they consistently reduced the abundance of zoonotic and/or pathogenic bacteria such as *Escherichia coli* and *Campylobacter* [88,94,105]. These results highlighted that antibiotics can profoundly modify the microbiota and in a variable way. Their use allows for beneficial effects on the abundance of zoonotic and/or pathogenic bacteria (Supplementary Data S2—Table S29).

#### 3.4.5. Plants and Seaweed

Only two studies examined the effect of the administration of plants in pigs. These two studies showed no change in the FCR [107,108], despite one of them showing an increase in the ADG [108]. The effect of these supplements on the abundance of *Lactobacillus* and SCFA-producing bacteria was inconsistent [107,108]. One of the controlled trials showed the beneficial effect of plants on the abundance of zoonotic and/or pathogenic bacteria by reducing the abundance of both *Salmonella* and *Escherichia coli* (Supplementary Data S2—Table S30).

### 3.5. Effect of Modulators on Intestinal Microbiota and Phenotype of Ruminants

#### 3.5.1. Antibiotics

Neomycin was found to increase body weight in calves [109]. The use of antibiotics in ruminants did not induce changes in α- or β-diversity [109,110]. One of the two studies demonstrated no modulation of the abundance of phyla in response to antibiotics [110]. The only study that investigated the abundance of lactic-acid-producing bacteria showed an increase in *Lactobacillus* and a change in the abundance of SCFA-producing bacteria *Akkermansia* and *Prevotella* [109]. These data predict that, in ruminants, antibiotics may also be able to induce a change in performance and in the composition of the gut microbiota, as has been shown in poultry and pigs (Supplementary Data S3—Table S31).

#### 3.5.2. Transplantation

Faecal transplantation into calves with diarrhoea induced an increase in body weight associated with a decrease in the rate of diarrhoea [109]. A change in β-diversity was associated with an increase in the abundance of *Lactobacillus* and a change in the abundance of SCFA-producing bacteria [109]. Rumen fluid transplantation induced a decrease in ADG without modifying the FCR [111]. Associated with a modification of β-diversity, a decrease in *Proteobacteria* abundance with rumen fluid from 3-month-old sheep and a decrease in *Actinobacteria* abundance with rumen fluid from 1-year-old sheep were observed [111]. These results demonstrate that the transfer of faecal or ruminal fluid can alter animal performance by modulating the composition of the microbiota (Supplementary Data S3—Table S32).

#### 3.5.3. Various Interventions

The administration of water at birth in calves did not induce any changes in the microbiota of the animals [112]. However, when colostrum was administered within 72 h of birth, an increase in the abundance of *Lactobacillus* and *Escherichia coli* was observed in the ileum and/or colon of calves [113] (Supplementary Data S3—Table S33).

## 4. Discussion

The data collected in this review reveal the great variability of microbiota response to modulators. Among the factors responsible for the variability, the choice of the animal genotype used is one of the most important. A specific study focused on the response of three chicken strains to capsicum/curcuma and demonstrated an opposite response of the ileal microbiota in Ross 308 broilers compared with the response observed in Cobb 500 and Hubbard broilers [31]. This variability of microbiota response depending on the host genotype has already been highlighted by numerous studies. In 2013, Zhao showed that 68 bacterial species were influenced by the chicken genotype, and that, of these 68 species, 15 belonged to the genus *Lactobacillus* [114]. Our approach was to highlight the deleterious or beneficial effect of families of modulators. However, within the same family, two modulators such as fructo-oligosaccharides and mannan-oligosaccharides can have diverging effects on the response of the microbiota [35]. Similarly, the gastrointestinal section studied is a source of variability. Of note, the composition of the microbiota varies from one gastrointestinal section to another, which is why it is not surprising that the response to a same modulator differs [60,95]. In addition to the factors described above, the techniques for studying microbiota, the dose of modulator, the duration of the intervention and the sex of the animals are also subject to variability. In this context, these variations make it difficult to draw a formal conclusion on the effect of certain modulators on the intestinal microbiota. Despite these variations, for certain modulators, the majority of studies showed a beneficial effect on both the phenotype and the microbiota. This was particularly the case for the use of probiotics and plants in poultry and the use of minerals and probiotics in pigs. In poultry, lipids, synbiotics and prebiotics acted on the intestinal microbiota without modifying the host phenotype. Some modulators had no effect on microbiota nor on phenotype: postbiotics and enzymes in poultry and prebiotics, amino acids and proteins in pigs. It appears that zoonotic and/or pathogenic agents are not safe to poultry and their microbiota. Data on the use of antibiotics demonstrated that, despite the beneficial effects on performance, a profound disturbance in the composition of the microbiota occurs without, however, highlighting any major deleterious changes. Nonetheless, in these articles, the absence of study of resistance genes to antibiotics should be underlined when considering these results. Indeed, the emergence of antibiotic-resistant genes in farms has been widely described and represents a major challenge [115,116,117,118]. These data therefore demonstrate that other microbiota modulators are able to increase animal performance as effectively as antibiotics without having deleterious effects on the composition of the gut microbiota. Finally, all of these data demonstrate that, depending on the species, the use of different families of modulators does not have the same effects on intestinal microbiota composition.

These results therefore provide information on the effect of certain modulators on both the intestinal microbiota and the host phenotype. However, in this type of study (controlled trials), the link between these two parameters is not developed. These studies are exclusively descriptive and therefore do not make it possible to confirm the correlation between variations in the gastrointestinal microbiota and the phenotypic changes in animals. Although limiting the study to controlled trials seemed to limit the information, particularly for ruminants, this choice attests to the quality of the studies taken into consideration. These studies therefore made it possible to obtain descriptive information on the effect of microbiota modulators on the composition of the gastrointestinal microbiota of farmed animals.

## 5. Conclusions

These results allow us to conclude on the effects of certain major families of modulators on the intestinal microbiota of poultry and pigs. Thus, the use of probiotics and plants in poultry, and the use of minerals and probiotics in pigs, appears as an interesting alternative to the use of antibiotics to increase performance. However, controlled trials have not collected enough information on ruminants, nor were they even able to establish links between microbiota modulation and physiological changes in the host. This highlights the need to perform more systematic studies in these important types of farm animals. Finally, concerning antibiotics, the lack of data on antibiotic resistance genes is also regrettable. However, limiting the study to controlled trials made it possible to certify the quality of the studies taken into consideration, which is important when it comes to studying parameters as variable as the response of the microbiota to modulators.

## Figures and Tables

**Table 1:** PICOS table for inclusion and exclusion criteria.

Parameter	Inclusion Criteria	Exclusion Criteria
Population	Poultry, pigs and ruminants Male and/or female	Other species
Intervention	Modulators administrated in feed, water or by gavage	Environmental intervention (i.e., temperature) or absence of intervention
Comparison	In physiological or pathological situation	No statistics
Outcomes	Quantitative study of the gut microbiota and/or pathogenic bacteria	No study of the microbiota. Qualitative studies of the gut microbiota
Study design	Controlled trials Systematic reviews	Review-type articles, in vitro or in silico studies, studies without placebo control group, consensus papers, letters to editor, book chapters, theses

## Data Availability

The web reports for the three species are available at: https://csbg.cnb.csic.es/RIMICIA/Poultry_p.html (accessed on 2 May 2023); https://csbg.cnb.csic.es/RIMICIA/Pigs_p.html (accessed on 2 May 2023); https://csbg.cnb.csic.es/RIMICIA/Ruminants_p.html (accessed on 2 May 2023).

## Supplementary Materials

The following are available online at https://www.mdpi.com/article/10.3390/microorganisms11061464/s1, Supplementary Data S1: Effect of Modulators on Intestinal Microbiota and Phenotype of Poultry (Table S1: Effect of probiotics on the phenotype and gastro-intestinal microbiota of poultry, Table S2: Effect of zoonotic/pathogenic bacteria on the phenotype and gastro-intestinal microbiota of poultry, Table S3: Effect of probiotics combined with another nutritional intervention on the phenotype and gastro-intestinal microbiota of poultry, Table S4: Effect of synbiotics on the phenotype and gastro-intestinal microbiota of poultry, Table S5: Effect of postbiotics on the phenotype and gastro-intestinal microbiota of poultry, Table S6: Effect of prebiotics on the phenotype and gastro-intestinal microbiota of poultry, Table S7: Effect of fibres on the phenotype and gastro-intestinal microbiota of poultry, Table S8: Effect of enzymes on the phenotype and gastro-intestinal microbiota of poultry, Table S9: Effect of enzymes combined with fibers on the phenotype and gastro-intestinal microbiota of poultry, Table S10: Effect of lipids on the phenotype and gastro-intestinal microbiota of poultry, Table S11: Effect of amino acids on the phenotype and gastro-intestinal microbiota of poultry, Table S12: Effect of antibiotics on the phenotype and gastro-intestinal microbiota of poultry, Table S13: Effect of antimicrobial peptides on the phenotype and gastro-intestinal microbiota of poultry, Table S14: Effect of plants and seaweed on the phenotype and gastro-intestinal microbiota of poultry, Table S15: Effect of minerals on the phenotype and gastro-intestinal microbiota of poultry, Table S16: Effect of polyphenol on the phenotype and gastro-intestinal microbiota of poultry, Table S17: Effect of vitamins on the phenotype and gastro-intestinal microbiota of poultry); Supplementary Data S2: Effect of Modulators on Intestinal Microbiota and Phenotype of Pigs (Table S18: Effect of minerals on the phenotype and gastro-intestinal microbiota of pigs, Table S19: Effect of polyphenol on the phenotype and gastro-intestinal microbiota of pigs, Table S20: Effect of immunoglobulin on the phenotype and gastro-intestinal microbiota of pigs, Table S21: Effect of probiotics on the phenotype and gastro-intestinal microbiota of pigs, Table S22: Effect of probiotics combined with other interventions on the phenotype and gastro-intestinal microbiota of pigs, Table S23: Effect of postbiotics on the phenotype and gastro-intestinal microbiota of pigs, Table S24: Effect of infection on the phenotype and gastro-intestinal microbiota of pigs, Table S25: Effect of prebiotics on the phenotype and gastro-intestinal microbiota of pigs, Table S26: Effect of fibres on the phenotype and gastro-intestinal microbiota of pigs, Table S27: Effect of amino acids on the phenotype and gastro-intestinal microbiota of pigs, Table S28: Effect of proteins on the phenotype and gastro-intestinal microbiota of pigs, Table S29: Effect of antibiotics on the phenotype and gastro-intestinal microbiota of pigs, Table S30: Effect of plants and seaweed on the phenotype and gastro-intestinal microbiota of pigs); Supplementary Data S3: Effect of Modulators on Intestinal Microbiota and Phenotype of Ruminants (Table S31: Effect of antibiotics on the phenotype and gastro-intestinal microbiota of ruminants, Table S32: Effect of transplantation on the phenotype and gastro-intestinal microbiota of ruminants, Table S33: Effect of various interventions on the phenotype and gastro-intestinal microbiota of ruminants). References [118,119,120,121,122,123,124,125] are cited in Supplementary Materials.
