# Supplementary material for: Effects of Major Families of Modulators on Performances and Gastrointestinal Microbiota of Poultry, Pigs and Ruminants: A Systematic Approach
Source: Microorganisms. 2023 May 31;11(6):1464. doi: 10.3390/microorganisms11061464 (PMC10300821; doi:10.3390/microorganisms11061464)
Supplement: Supplementary file 1 [file microorganisms-11-01464-s001.zip › Supplementary Data S1.pdf]

Supplemental Data S1: Effect of Modulators on Intestinal Microbiota and Phenotype of Poultry

Table S1: Effect of probiotics on the phenotype and gastro-intestinal microbiota of poultry

Ref	Intervention	Conditions	Methods	Phenotype			Immunity	Microbiota			
				**Weight**	**Food intake**	**FCR or FCE**		**Diversity**	**Lactic acid-producing bacteria**	**SCFA-producing bacteria**	**Pathogenic and/or Zoonotic bacteria**
Rodjan 2017	*Bacillus*	Ross 308 broiler chickens<br>Male and Female<br>Healthy	*Bacillus* (5.10^{11} CFU/kg of diet)<br>5 weeks<br>Culture	=	=	=	ND	ND	Ileum and Cecum:<br>= Lactobacillus	ND	Ileum and Cecum:<br>↓E.coli
Cao 2018	*Bacillus Amyloliquefaciens*	Ross 308 broiler chickens<br>Gender ND<br>Healthy	*Bacillus* (10^{9} CFU/ kg of diet)<br>6 weeks<br>Sequencing	↑ BW<br>↑ BWG	ND	↓ FCR	ND	ND	Cecum:<br>= Lactobacillus	Caecum:<br>↑ Faecalibacterium<br>↑ Blautia	ND
Xing 2015	*Bacillus Subtilis*	Linwu ducklings<br>Female<br>Healthy	*Bacillus* (5.10^{8} or 5.10^{10} CFU /kg of diet)<br>9 weeks<br>RTqPCR	ND	ND	ND	Jejunum:<br>↑ INFγ mRNA levels<br>↓ IL-1β, IL-2, IL-10, IL-18 mRNA levels<br>Ileum:<br>↓ IL-1β, IL-2, IL-10, IL-8 mRNA levels	ND	Caecum:<br>↑ Lactobacillus<br>= Bifidobacterium	Caecum:<br>↑ Megasphaera elsdenii	Cecum:<br>= E.coli
Jeong 2014	*Bacillus Subtilis*	Ross 308 broiler chickens<br>Male<br>Healthy	*Bacillus* (3.10^{8} or 6.10^{8} CFU/kg of diet)<br>5 weeks<br>Culture	↑ ADG	=	↓ FCR	ND	ND	Ileum, Cecum and feces:<br>↑ Lactobacillus	ND	Cecum and Feces:<br>↓ E.coli<br>Cecum, Ileum, Colon and Feces:<br>↓ Salmonella<br>= C.perfringens
Pereira 2018	*Bacillus Subtilis*	Cobb 500 broiler chickens<br>Male<br>Healthy	*Bacillus* (3.10^{7} CFU/kg of diet)<br>6 weeks<br>Sequencing	=	=	=	ND	↓ α-diversity and richness	Small intestine:<br>↑ Lactobacillus	Small intestine:<br>↓Faecalibacterium	ND
Chen 2013	*Bacillus Subtilis + Saccharomyces cerevisiae*	Landes geese<br>Male<br>Healthy	*Bacillus* (10^{9} CFU/kg of diet)<br>3 weeks<br>Culture	↑ BWG	↑	=	↑[GPx] in heart and liver<br>↑ [SOD] in meat<br>↓ [MDA] in heart, liver and meat	ND	Large and small intestine:<br>↑ Lactobacillus	ND	Small and Large intestine:<br>↓ E.coli
Han 2013	*Enterococcus fecalis*	Arbor Acres broiler chickens<br>Male and Female<br>Healthy	*Enterococcus* (10^{9}, 10^{10} or 10^{11} CFU/kg of diet)<br>6 weeks<br>RTqPCR	↑ BW<br>↑ ADG	=	↓ FCR	↓ [MDA] in plasma	= α-diversity, eveness and richness	Feces:<br>↑ Lactobacillus<br>↑ Bifidobacterium	ND	Cecum:<br>= E.coli
Yang 2012	*Clostridium butyricum*	Lignan yellow broiler chicken<br>Male<br>Healthy	*Clostridium* (1 to 3 10^{7} CFU/kg of diet)<br>6 weeks<br>Culture	↑ BW<br>↑ ADG	ND	ND	↑ [IgA] [IgG] and [IgM] in serum	ND	Cecum:<br>↑ Lactobacillus<br>↑ Bifidobacterium	ND	Cecum:<br>↓ E.coli<br>↓ Salmonella<br>↓ C.perfringens
Vase-Khavari 2018	Superzist	Ross 308 broiler chickens<br>Gender ND<br>Healthy	Superzist (0.03% w/v of diet)<br>6 weeks<br>Culture	=	=	=	↑ [Lymphocyte] in plasma	ND	Cecum:<br>= Lactobacillus	ND	Cecum:<br>= E.coli
Attebury 2011	*Bdellovibrio Bacteriovorus*	Hy-line Brown chicken<br>Male<br>Healthy	*Bdellovibrio* (1.9 10^{7} PFU/mL ; 100µL/animal/day)<br>Salmonella (3.16 10^{7} CFU/animal)<br>4 weeks<br>Culture	ND	ND	ND	ND	ND	Cecum:<br>↓ Lactobacillus<br>= Bifidobacterium	ND	ND
		Hy-line Brown chicken<br>Male<br>S. Enteriditis inoculation		ND	ND	ND	ND	ND	ND	ND	Cecum:<br>↓ Salmonella
Wang 2021 bis	*Lactobacillus salivarius*	White leghorn chicken<br>Gender ND<br>Mycoplasma gallisepticum inoculation	*Lactobacillus* (5.10^{7} CFU/animal/day)<br>Mycoplasma (10^{9} CCU/mL ; 0.2 mL/animal)<br>1 week<br>Sequencing	ND	ND	ND	ND	↑ α-diversity	ND	ND	Feces:<br>↓ E.coli

Table S2: Effect of zoonotic/pathogenic bacteria on the phenotype and gastro-intestinal microbiota of poultry

Ref	Intervention	Conditions	Methods	Phenotype				Immunity	Intestinal barrier	Microbiota			
				Weight	Food intake	FCR or FCE	Villi structure			Diversity	Phylum	Lactic acid-producing bacteria	Pathogens
Yang 2019	*C. perfringens*	Cobb broiler chicken<br>Male and Female	Infection (2.5 10^8 CFU/mL ; 3mL/animal)<br>3 weeks<br>Sequencing	ND	ND	ND	ND	ND	ND	Jejunum and Cecum:<br>= α-diversity	Jejunum:<br>↓ Cyanobacteria<br>↓ Bacteroidetes<br>↑ Proteobacteria	Jejunum:<br>↓ Lactobacillus<br>Caecum:<br>= Lactobacillus	Jejunum and Cecum:<br>= Escherichia shigella<br>= Clostridium-sensus stricto 1
	Eimera			ND	ND	ND	ND	ND	ND	Jejunum and Cecum:<br>= α-diversity	Jejunum:<br>↓ Firmicutes<br>↓ Bacteroidetes<br>↑ Cyanobacteria	Jejunum:<br>= Lactobacillus<br>Caecum:<br>= Lactobacillus	Jejunum and Cecum:<br>= Escherichia shigella<br>= Clostridium-sensus stricto 1
	C.perfringens + Eimera			ND	ND	ND	ND	ND	ND	Jejunum:<br>↓ α-diversity<br>Caecum:<br>= alpha diversity	Jejunum:<br>↓ Cyanobacteria<br>↓ Actinobacteria<br>↑ Proteobacteria	Jejunum:<br>= Lactobacillus<br>Caecum:<br>= Lactobacillus	Jejunum and Cecum:<br>= Escherichia shigella<br>Jejunum:<br>↑ Clostridium-sensus stricto 1
Kim 2015	*C.perfringens* + E.maxima	Ross 308 broiler chickens<br>Male	*C.perfringens* (10^9 CFU/animal)<br>E.maxima (10^4 oocysts/animal)<br>3 weeks<br>Sequencing	↓ BW	ND	ND	ND	ND	ND	ND	ND	Ileum:<br>↓ Lactobacillus	ND
		Cobb 500 broiler chickens<br>Male		↓ BW	ND	ND	ND	ND	ND	ND	ND	Ileum:<br>↑ Lactobacillus	ND
		Hubbard broiler chickens<br>Male		↓ BW	ND	ND	ND	ND	ND	ND	ND	Ileum:<br>↓ Lactobacillus	ND
Yang 2008	*E.coli*	Cobb 500 broiler chickens<br>Male	E.coli (10^7 CFU/ml ; 1mL/animal then 10^5 CFU/mL of water)<br>3 weeks<br>Culture	= BW	↑	↑ FCR	ND	ND	ND	ND	ND	Ileum:<br>↑ Lactobacillus	ND
Daneshmand 2019	*E.coli*	Cobb broiler chicken<br>Male	*E.coli* (10^8 CFU/animal)<br>3 weeks<br>Culture	↓ ADG	=	↑ FCR	Jejunum:<br>↓ VH, VW and VSA<br>= CD and VH/CD ratio	Jejunum:<br>↑ [IL-2]<br>↓ [IL-6]	Jejunum:<br>↓ [Claudin-1] and [Occludin]<br>↑ [MUC2]	ND	ND	Ileum:<br>↓ Lactobacillus<br>↓ Bifidobacterium	Ileum:<br>↑ E.coli
Shao 2014	*Salmonella thyphimurium*	Cobb 500 broiler chicken<br>Male	*Salmonella* (109 CFU/mL ; 0.8 mL/bird)<br>2 or 6 weeks<br>RTqPCR	↓ BW	=	=	Ileum:<br>= VH<br>= CD<br>↓ VH/CD ratio	ND	ND	Ileum:<br>↓ α-diversity<br>= richness	ND	Ileum:<br>↓ Lactobacillus<br>= Enterococcus	Ileum:<br>↑ Salmonella Typhimurium
Wang 2021	*Salmonella pullorum*	Dward chicken<br>Male	*Salmonella* (8.45$10^8$ CFU/mL ; 0.2 mL/bird)<br>11 days<br>Culture	↓ BW<br>↓ADG	↓	=	Ileum:<br>↓ VH and regularity	Cecum:<br>↓ [IgA]<br>= [IL-4]<br>↑ [TNFα]<br>↑ [INFγ]<br>↑ Lymphocyte infiltration	ND	ND	ND	Cecum:<br>↓ Lactobacillus	Cecum:<br>↑ E.coli<br>↑ Salmonella
Fernandez 2002	*Salmonella enteritidis*	Isa-brown laying hens<br>Female	*Salmonella* (5$10^4$ CFU/mL)<br>4 weeks<br>Culture	ND	ND	ND	ND	ND	ND	ND	ND	Cecum:<br>= Lactobacillus<br>= Enteroccocus	Cecum:<br>↑ Salmonella enteriditis
Wang 2021 bis	*Mycoplasma gallispeticum*	White Leghorn Chicken	*Mycoplasma* (10^9 CFU/mL ; 0.2 mL/bird)<br>1 week<br>Sequencing	ND	ND	ND	ND	Ileum:<br>↓ [SIgA]<br>↓ Muc2 mRNA	Ileum:<br>↓ transepitheliale electrical reistance<br>↑ FD4 flux<br>↓ Claudine-1, Occludin and ZO-1	Feces:<br>↓ α-diversity<br>Modification of the β-diversity	Feces:<br>↑ Firmicutes<br>↑ Proteobacteria<br>↓ Bacteroidetes<br>↓ Actinobacteria	ND	Feces:<br>↑ E.coli

Table S3: Effect of probiotics combined with another nutritional intervention on the phenotype and gastro-intestinal microbiota of poultry

Ref	Probiotic	Intervention	Conditions	Methods	Phenotype				Immunity	Microbiota		
					Weight	Food intake	FCR or FCE	Villi structure		Diversity	Lactic acid-producing bacteria	Pathogenic and/or Zoonotic bacteria
Rodjan 2017	*Bacillus*	Organic acids:<br>Fummaric acid + Formic acid + Lactic acid + Propionic acid + Citric acid	Ross 308 broiler chickens<br>Male and Female<br>Healthy	*Bacillus* (5.10^{11} CFU/kg of diet)<br>Organic acids (2g /kg of diet)<br>5 weeks<br>Culture	=	=	=	Duodenum:<br>= VH, CD and VH/CD ratio	ND	ND	Ileum and Cecum<br>= Lactobacillus	Ileum and Cecum:<br>↓ E.coli
Wang 2021	*Lactobacillus paracasei and Plantarum*	Fermented herbs:<br>Astragalus + P. notoginseng + Licorice + Chickpeas + Black bean powder + Glucose +	Dward chicken<br>Male<br>Healthy	*Lactobacillus* ($1.2\ 10^9$ CFU/mL ; 0.2 mL/animal)<br>Herbs (10% in water)<br>*Salmonella* ($8.45\ 10^8$ CFU/mL ; 0.2mL/animal)<br>11 days<br>Culture	↑ BW<br>↑ADG	↑	=	= (Ileum)	Cecum:<br>= IgA and IL-4<br>↓ Lymphocyte infiltration<br>↓ TNFα and INFγ	ND	Cecum:<br>↑ Lactobacillus	Cecum:<br>↓ E.coli<br>↓ Salmonella
			Dward chicken<br>Male<br>*Salmonella pullorum*		↑ BW<br>↑ADG	↑	=	Ileum:<br>↑ VH and regularity	Cecum:<br>↑ IgA and IL-4<br>↓ TNFα<br>= Lymphocyte infiltration	ND	Cecum:<br>↑ Lactobacillus	Cecum:<br>↓ E.coli<br>↓ Salmonella
Huang 2020	*Bacillus licheniformis*	Fermented herbs:<br>Codonopsis Radix, Astragali Radix, Radix Glycyrrhizae, Poria, Edicated Leaven, Malt, Cra taegu, Areca Seed	Arbor acre plus broiler chicken<br>Male<br>Healthy	Fermented Herbs by probiotic (20 mg/kg of diet)<br>6 weeks<br>Sequencing	=	=	=	ND	ND	= (Fogut)	Forgut<br>= Lactobacillus<br>= Bifidobacterium	ND
	Bacillus subtilis				=	=	=	ND	ND	= (Fogut)	Forgut<br>= Lactobacillus<br>= Bifidobacterium	ND
	Saccharomyces cerevisae stain 1				=	=	=	ND	ND	= (Fogut)	Forgut<br>= Lactobacillus<br>= Bifidobacterium	ND
	Zygosaccharo myces rouxii				=	=	=	ND	ND	= (Fogut)	Forgut<br>↓ Lactobacillus<br>↑ Bifidobacterium	ND
	Saccharomyces cerevisiae strain 2				=	=	=	ND	ND	= (Fogut)	Forgut<br>= Lactobacillus<br>= Bifidobacterium	ND
Chen 2021	*Bacillus subtilis*	Fermented product	Ross 308 broiler chicken<br>Male<br>LPS challenge	*Bacillus* (2 or 6 10^{10} CFU/kg of diet)<br>LPS (5mg/kg of BW)<br>3 weeks<br>Sequencing	=	=	=	ND	↓ IL-6, IL-1β (duodenum, jejunum and Ileum)	↓ α-diversity<br>Modification of β-diversity	Cecum<br>= Lactobacillus<br>= Bifidobacterium	ND

Table S4: Effect of synbiotics on the phenotype and gastro-intestinal microbiota of poultry

Ref	Probiotic	Prebiotic	Conditions	Methods	Phenotype				Immunity	Intestinal barrier	Microbiota		
					**Weight**	**Food intake**	**FCR or FCE**	**Villi structure**			**Lactic acid-producing bacteria**	**AGCC**	**Pathogenic and/or Zoonotic bacteria**
Vineetha 2016	*Lactobacillus plantarum*	MOS	Aribro Dhanraja broiler chicken Healthy	Probiotic (10^8 CFU/g of fermented diet) Prebiotic (1g/kg of diet) 5 weeks Culture	=	=	=	Duodenum ↑VH ↑CD = VH/CD ratio	ND	ND	Crop and Ileum: ↑ Lactobacillus	ND	Crop, Ileum and Cecum: ↓ E.coli ↓ Salmonella
	Lactobacillus acidophilus				=	=	=	Duodenum ↑VH ↑CD = VH/CD ratio	ND	ND	Crop, Ileum and Cecum: ↑ Lactobacillus	ND	Crop, Ileum and Cecum: ↓ E.coli ↓ Salmonella
Markazi 2018	Poultrystar ®		White Leghorn Chicken *Salmonella enteritidis*	Synbiotic (20 mg/day) Salmonella (10^9 CFU/animal) 29 weeks RTqPCR	=	ND	ND	= VH (jejunum)	Bile and plasma : ↑ IgA	ND	Cecum: ↑ Lactobacillus ↑ Bifidobacterium ↑Pediococcus acidilacti	ND	Cecum: ↓ Salmonella
Mountzouris 2019	Poultrystar ®		Cobb broiler chicken Male Healthy	Synbiotic (1g/kg of diet) 6 weeks RTqPCR	ND	ND	ND	ND	= (Ileum)	= (Ileum)	Ileum and Cecum: = Lactobacillus = Bifidobacterium	= (Ileum and Caecum)	Ileum and Cecum: = E.coli = C.perfringens
	Thermal inactivated Poultrystar ®				ND	ND	ND	ND	= (Ileum)	= (Ileum)	Ileum and Cecum: = Lactobacillus = Bifidobacterium	= (Ileum and Caecum)	Ileum and Cecum: = E.coli = C.perfringens

Table S5: Effect of postbiotics on the phenotype and gastro-intestinal microbiota of poultry

Ref	Intervention	Conditions	Methods	Phenotype				Microbiota				
				Weight	Food intake	FCR or FCE	Villi structure	Lactic acid-producing bacteria	SCFA-producing bacteria	pH	AGCC	Pathogenic and/or Zoonotic bacteria
Rodjan 2017	Fummaric acid + Formic acid + Lactic acid + Propionic acid + Citric acid	Ross 308 broiler chickens<br>Male and Female<br>Healthy	Organic acids (2g /kg of diet)<br>5 weeks<br>Culture	=	=	=	= (Duodenum)	Ileum and Cecum:<br>= Lactobacillus	ND	ND	ND	↓E.coli (Ileum and Caecum)
Nava 2009	HLP: Batanoïc, Lactic and Phosphoric acid	Cobb broiler chickens<br>Male<br>Healthy	HLP (0.0525% of water)<br>4 weeks<br>RTqPCR	=	=	=	ND	Ileum:<br>= Lactobacillus	ND	ND	ND	ND
	HFP: Butanoïc, Formic and Proprionic acid		HFP (0.0525% of water)<br>4 weeks<br>RTqPCR	=	=	=	ND	Ileum:<br>↑ Lactobacillus	ND	ND	ND	ND
Czerwinski 2012	Sodium Butyrate	Ross 308 broiler chickens<br>Female<br>Healthy	SB (300 mg/kg of diet)<br>4 weeks<br>FISH probing	=	=	=	= (Jejunum and Ileum)	Ileum and Cecum:<br>= Lactobacillus<br>= Enterococcus	ND	↑<br>(Ileum)	Ileum:<br>↑ [Proprionate]<br>↑ [Isobutyrate]<br>= [Actetate]<br>= [Lactic acid]	ND
Feng 2021	Monobutyrin	Qingyan Partridge broilers<br>Female<br>Healthy	MB (250 mg/kg of diet)<br>8 weeks<br>Sequencing	↑ (Egg weight)	ND	ND	ND	Cecum:<br>= Lactobacillus	Cecum:<br>= Faecalibacterium, Phascolarctobacterium	ND	ND	ND

Table S6: Effect of prebiotics on the phenotype and gastro-intestinal microbiota of poultry

				Phenotype				Microbiota			
**Ref**	**Intervention**	**Conditions**	**Methods**	**Weight**	**Food intake**	**FCR or FCE**	**Villi structure**	**Lactic acid-producing bacteria**	**pH**	**SCFA**	**Pathogenic and/or Zoonotic bacteria**
Peinado 2013	Inulin	Cobb broilers chickens<br>Male<br>Healthy	Inulin (20g/kg of diet)<br>3 weeks<br>RTqPCR	=	=	=	Ileum<br>↑VH<br>= CD<br>↑VH/CD ratio	Crop, Ileum and Cecum:<br>↓ Lactobacillus<br>= Bifidobacterium	= (crop)	ND	Crop, Ileum and Cecum:<br>= Escherichia-shigella<br>= Enterobacteria
Dias 2019	Inulin	Cornish Cross broiler<br>Male and Female<br>Healthy	Inulin (40mg per egg)<br>3 weeks<br>RTqPCR	=	ND	ND	ND	Cecum:<br>↑ Lactobacillus<br>↑ Bifidobacterium	ND	ND	Caecum:<br>↑ E.coli
Kulshreshtha 2014	Inulin	Lohmann Brown Classic Laying Hens<br>Female<br>Healthy	Inulin (2% of diet)<br>4 weeks<br>RTqPCR	=	=	=	= (ileum)	Ileum:<br>↑ Lactobacillus acidophilus<br>= Bifidobacterium Longum	ND	Cecum:<br>↑ [i-butyric acid]<br>= [acetate], [proprionate] and [butyrate]	Ileum:<br>↓ C.perfringens
Rebolé 2010	Inulin	Cobb broilers chickens<br>Male<br>Healthy	Inulin (10 or 20g of diet)<br>5 weeks<br>Culture	=	=	=	= (jejunum)	Ileum and Cecum:<br>↑ Lactobacillus<br>↑ Bifidobacterium	= (cecum)	Cecum:<br>= [acetate], [proprionate] and [butyrate]	ND
Shang 2010	Inulin	Brown nick laying hens<br>Female<br>Healthy	Inulin (0.1 to 2% of diet)<br>4 weeks<br>Culture	ND	=	=	ND	Cecum:<br>= Lactobacillus<br>↑ Bifidobacterium	↓(cecum)	ND	ND
Xia 2019	Inulin	Tegek broiler chickens<br>Male<br>Healthy	Inulin (1 to 4% of diet)<br>6 weeks<br>FISH probing	↑ BW (4%)<br>↑ BWG (2%)	↓ (2%)	=	ND	Cecum:<br>↑ Lactobacillus<br>↑ Bifidobacterium	ND	ND	Cecum:<br>↑ Alpha proteobacteria
Yang 2008	MOS	Cobb broilers chickens<br>Male<br>Healthy	MOS (2g/kg of diet)<br>3 weeks<br>Culture	=	=	=	= (jejunum)	Ileum:<br>↑ Lactobacillus	= (jejunum, Ileum, Cecum)	Ileum:<br>= [Lactic acid] and [acetate]	Jejunum, Ileum and Cecum:<br>= C.perfringens
	MOS	Cobb broilers chickens<br>Male<br>E.coli inoculation		=	=	↑ FCR	= (jejunum)	Jejunum, Ileum, Ceacum:<br>= Lactobacillus	= (jejunum, Ileum, Cecum)	Ileum:<br>= [Lactic acid] and [acetate]	Jejunum, Ileum and Cecum:<br>= C.perfringens
	FOS	Cobb broilers chickens<br>Male<br>Healthy	FOS (10g/kg of diet)<br>3 weeks<br>Culture	=	↑	=	= (jejunum)	Ileum:<br>↑ Lactobacillus	↓(ileum)	Ileum:<br>↑ [Lactic acid]<br>=[acetate]	Jejunum, Ileum and Cecum:<br>= C.perfringens
	FOS	Cobb broilers chickens<br>Male<br>E.coli inoculation		↑ BWG	=	=	= (jejunum)	Jejunum, Ileum, Cecum:<br>= Lactobacillus	= (jejunum, Ileum, Cecum)	Ileum:<br>↑ [Lactic acid]<br>=[acetate]	Cecum:<br>↓ C.perfringens
Fernandez 2002	MOS	Isa-brown laying hens<br>Female<br>Healthy	MOS (2.5% of diet)<br>4 weeks<br>Culture	ND	ND	ND	ND	Cecum:<br>= Lactobacillus<br>= Bifidobacterium<br>↑ Enterococcus	ND	ND	Cecum:<br>↓ S.enteriditis
		Isa-brown laying hens<br>Female<br>Salmonella enteriditis		ND	ND	ND	ND	Cecum:<br>= Lactobacillus<br>= Enterococcus	ND	ND	Cecum:<br>↓ S.enteriditis

Table S7: Effect of fibres on the phenotype and gastro-intestinal microbiota of poultry

Ref	Intervention	Conditions	Methods	Phenotype				Microbiota	
				**Weight**	**Food intake**	**FCR or FCE**	**Villi structure**	**Lactic acid-producing bacteria**	**Pathogenic and/or Zoonotic bacteria**
Xie 2020	Locust bean gum	AA broiler chicken Healthy	Fiber (0.1% of diet)<br>6 weeks<br>FISH probing	ND	ND	ND	Duodenum, Jejunum and Ileum:<br>= VH<br>=CD<br>=VH/CD ratio	Cecum:<br>= Lactobacillus	Cecum:<br>= E.coli
	Locust bean gum enzymolysate		Fiber (0.01% to 0.03% of diet)<br>6 weeks<br>FISH probing	ND	ND	ND	Duodenum and Ileum:<br>↑ VH<br>= CD<br>Jejunum:<br>= VH<br>= CD	Cecum:<br>↑ Lactobacillus	Cecum:<br>↓ E.coli
Venardou 2021	Laminarin	Ross 308 broiler chicken Male Healthy	Fiber (150 to 300 ppm)<br>5 weeks<br>Sequencing	↑ BW<br>↑ BWG	↑	=	ND	Cecum:<br>= Lactobacillus<br>↑ Bifidobacterium	ND
Pandi 2017	Sweet patato flour	Cobb broiler chicken Male Healthy	5 weeks<br>Culture	↓ BW<br>↓ BWG	↑	↑	Jejunum and Ileum<br>= VH<br>= CD<br>= VH/CD ratio	Cecum:<br>= Lactobacillus	Cecaum:<br>= E.coli
Fernandez 2002	PKM (contain xylan, arabinans, galactans…)	Ise brown laying hen Female Healthy	Fiber (2.5% of diet)<br>4 weeks<br>Culture	ND	ND	ND	ND	Cecum:<br>= Lactobacillus<br>↑ Bifidobacterium<br>↓ Enterococcus	Caecum:<br>↓ Salmonella
Peinado 2013	di-D-frutose dianhydride-enriched caramels	Cobb broilers chickens Male Healthy	Fiber (20g/kg of diet)<br>3 weeks<br>RTqPCR	↑ BW<br>↑ BWG	↑	=	Ileum<br>= VH<br>= CD<br>= VH/CD ratio	Crop, ileum and Cecum:<br>= Lactobacillus<br>= Bifidobacterium	Crop and Cecum:<br>↓ Escherichia-Shigella

Table S8: Effect of enzymes on the phenotype and gastro-intestinal microbiota of poultry

Ref	Intervention	Conditions	Methods	Phenotype				Microbiota		
				**Weight**	**Food intake**	**FCR or FCE**	**Villi structure**	**Lactic acid-producing bacteria**	**pH**	**Pathogenic and/or Zoonotic bacteria**
Pandi 2017	Endo-1, 3 9 (4) β-glucanase<br>+<br>Endo-1, 4 β-xylanase	Cobb broiler chicken<br>Male<br>Healthy	5 weeks<br>Culture	=	=	=	Jejunum and Ileum:<br>= VH<br>= CD<br>= VH/CD ratio	Cecum:<br>= Lactic acid bacteria	ND	Cecum:<br>= E.coli
Rebolé 2010	Endo-1,4-β-glucanase<br>+<br>Endo-1, 3 (4) β-glucanase<br>+<br>Endo-1,4 β-xylanase	Cobb broiler chicken<br>Male<br>Healthy	Enzyme (100 mg/kg of diet)<br>5 weeks<br>Culture	=	=	=	Jejunum:<br>= VH<br>= CD<br>= VH/CD ratio	Ileum and cecum:<br>= Lactobacillus<br>= Bifidobacterium	= (cecum)	ND
Shakouri 2008	Endo-1,4 β-glucanase<br>+<br>Endo-1,4 β-xylanase	Cobb broiler chicken<br>Male<br>Healthy<br>Diet 1 (Barley)	Enzyme (0.05% of diet)<br>4 weeks<br>Culture	=	=	=	Jejunum:<br>= VH<br>↑ CD<br>= VH/CD ratio	Gizzard, Ileum and Cecum:<br>= Lactobacillus	= (gizzard, ileum and caecum)	Gizzard, Ileum and Cecum:<br>= C. perfringens
		Cobb broiler chicken<br>Male<br>Healthy<br>Diet 2 (Maize)		=	=	=	Jejunum:<br>= VH<br>= CD<br>= VH/CD ratio	Gizzard, Ileum and Cecum:<br>= Lactobacillus	= (gizzard, ileum and cecum)	Gizzard, Ileum and Cecum:<br>= C. perfringens
		Cobb broiler chicken<br>Male<br>Healthy<br>Diet 3 (Sorghum)		=	=	=	Jejunum:<br>= VH<br>↓ CD<br>= VH/CD ratio	Gizzard:<br>↑ Lactobacillus<br>Ileum and caecum<br>= Lactobacillus	= (gizzard, ileum and cecum)	Gizzard, Ileum and Cecum:<br>= C. perfringens
		Cobb broiler chicken<br>Male<br>Healthy<br>Diet 4 (Wheat)		↓ BWG	↓	=	Jejunum:<br>↑ VH<br>= CD<br>↑VH/CD ratio	Gizzard:<br>↑ Lactobacillus<br>Ileum and caecum<br>= Lactobacillus	= (gizzard, ileum and cecum)	Gizzard, Ileum and Cecum:<br>= C. perfringens
Ptak 2015	Phytase	Ross 308 broilers chicken<br>Female<br>Healthy	Enzyme (5000 FTU/kg of diet)<br>4 weeks<br>FISH probing	↑ BWG	=	↓ FCR	ND	Ileum:<br>↑ Lactobacillus / Enterococcus<br>↓ Streptococcus / Lactococcus<br>↓ Bifidobacterium	↑ (crop)<br>= (ileum and caecum)	Ileum:<br>= C. perfringens
Wu 2017	Phytase	Ross 308 broilers chicken<br>Female<br>Healthy<br>Diet 1 (Wheat)	Enzyme (1000 FTU/kg of diet)<br>4 weeks<br>RTqPCR	↑ BWG	↑	=	ND	Cecum:<br>= Lactobacillus<br>= Enterococcus	ND	ND
		Ross 308 broilers chicken<br>Female<br>Healthy<br>Diet 2 (Maize)		↑ BWG	↑	=	ND	Cacum:<br>= Lactobacillus<br>= Enterococcus	ND	ND

Table S9: Effect of enzymes combined with fibers on the phenotype and gastro-intestinal microbiota of poultry

					Phenotype				Microbiota			
**Ref**	**Enzyme**	**Fiber**	**Conditions**	**Methods**	**Weight**	**Food intake**	**FCR or FCE**	**Villi structure**	**Lactic acid-producing bacteria**	**pH**	**SCFA**	**Pathogenic and/or Zoonotic bacteria**
Pandi 2017	Endo-1, 3 9 (4) β-glucanase<br>+<br>Endo-1, 4 β-xylanase	Sweet patato flour	Cobb broiler chicken<br>Male<br>Healthy	5 weeks<br>Culture	↓ BW<br>↓ BWG	↑	↑ FCR	Jejunum and Ileum:<br>= VH<br>= CD<br>= VH/CD ratio	Cecum:<br>= Lactic acid bacteria	ND	ND	Cecum:<br>= E.coli
Rebolé 2010	Endo-1,4-β-glucanase<br>+<br>Endo-1, 3 (4) β-glucanase<br>+<br>Endo-1,4 β-xylanase	Inulin	Cobb broiler chicken<br>Male<br>Healthy	Enzyme (100 mg/kg of diet)<br>Prebiotic (20g/kg of diet)<br>5 weeks<br>Culture	=	=	=	Jejunum:<br>= VH<br>= CD<br>= VH/CD ratio	Ileum and Cecum:<br>= Lactobacillus<br>= Bifidobacterium	= (cecum)	Cecum:<br>= [Total SCFA], [Lactic acid], [Propionate], [Acetate] and [Butyrate]	ND

Table S10: Effect of lipids on the phenotype and gastro-intestinal microbiota of poultry

				Phenotype					Microbiota					
**Ref**	**Intervention**	**Conditions**	**Methods**	**Weight**	**Food intake**	**FCR or FCE**	**Villi structure**	**Immunity**	**Diversity**	**Phylum**	**Lactic acid-producing bacteria**	**SCFA-producing bacteria**	**SCFA**	**Pathogenic and/or Zoonotic bacteria**
Wu 2021	Lauric acid	Ross 308 broiler chicken<br>Male<br>Healthy	LA (500 or 1000 mg/kg of diet)<br>6 weeks<br>Sequencing	↑ BW<br>↑ BWG	↑ FI	=	Duodenum and Ileum:<br>↑ VH<br>↓ CD<br>↑ VH/CD ratio	Serum:<br>↑ [IgA],[IgM] and [IgY]<br>↓ [TNFα], [IL-4] and [IL-10]	Cecum:<br>= α-diversity	Cecum<br>= phylum abundance	ND	Cecum:<br>↓ Phascolarctobacterium<br>↑ Faecalibacterium	Cecum:<br>↓ [Acetate], [Butyrate] and [Propionate]	ND
Yang 2019	Lauric acid	Cobb broiler chicken<br>Male and Female<br>C.perfringens + Eimera	LA (400 mg/ kg of diet)<br>3 weeks<br>Sequencing	ND	ND	ND	ND	ND	Jejunum:<br>↓ α-diversity<br>Cecum:<br>= α-diversity	Jejunum:<br>↓ Proteobacteria<br>↓ Cyanobacteria<br>↓ Bacteroidetes<br>↑ Firmicutes	Cecum and Jejunum:<br>= Lactobacillus	ND	ND	Cecum and Jejunum:<br>= Escherichia shigella<br>Jejunum:<br>↑ Clostridium sensus stricto 1
Feng 2021	Glycerol monolaurate	Qingyuan Patridge broilers<br>Female<br>Healthy	GM (300 mg/ kg of diet)<br>8 weeks<br>Sequencing	ND	ND	ND	ND	ND	Cecum:<br>= α-diversity	Cecum:<br>↓ Proteobacteria	Cecum:<br>= Lactobacillus<br>= Streptococcus	Cecum:<br>= Phascolarctobacterium<br>= Feacalibacterium<br>= Bacteroides	ND	ND

Table S11: Effect of amino acids on the phenotype and gastro-intestinal microbiota of poultry

				Phenotype					Microbiota		
**Ref**	**Intervention**	**Conditions**	**Methods**	**Weight**	**Food intake**	**FCR or FCE**	**Villi structure**	**Immunity**	**Phylum**	**Lactic acid-producing bacteria**	**Pathogenic and/or Zoonotic bacteria**
Ruan 2020	L-arginine	Quingyan chicken<br>Female<br>Healthy	AA (9.7 to 13.3 g/kg of diet)<br>4 weeks<br>Sequencing	↑ BW<br>↑ BWG	↑ FI<br>↑ ADFI	↓ FCR	ND	Blood:<br>↑ % lymphocyte CD3+, CD4+ and CD4+/CD8+ ratio<br>Jejunum and ileum:<br>↑ [GSH-Px],[T-AOC], [HMOX1] and [NRF2]<br>↓ [MDA] jejunum (diminution du Stressox)<br>Ileum:<br>↑ [GPx1] and [sIgA]<br>↓ [IL-1β], [MYD88] and [TLR4]<br>Jejunum:<br>↑ [IgG]	Ileum:<br>↑ Firmicutes<br>↓ Proteobacteria	Ileum:<br>= Lactobacillus<br>= Enterococcus	Ileum:<br>= Escherichia-shigella<br>↓ Clostridium sensus stricto 1

Table S12: Effect of antibiotics on the phenotype and gastro-intestinal microbiota of poultry (1/2)

Ref	Intervention	Conditions	Methods	Phenotype				Immunity	Intestinal barrier	Microbiota						
				Weight	Food intake	FCR or FCE	Villi structure			Diversity	Phylum	Lactic acid-producing bacteria	SCFA-producing bacteria	pH	SCFA	Pathogenic and/or Zoonotic bacteria
Nava 2009	Bacitracin	Cobb broiler chicken<br>Male<br>Healthy	Antibiotic (25ppm of diet)<br>4 weeks<br>RTqPCR	=	=	=	ND	ND	ND	ND	ND	Ileum:<br>= Lactobacillus	ND	ND	ND	ND
Vineetha 2016	Bacitracin	Aribro Dhanraja broiler chicken<br>Healthy	Antibiotic (20g/100g of diet)<br>5 weeks<br>Culture	=	=	=	Duodenum:<br>↓ VH<br>↓ CD<br>= VH/CD ratio	ND	ND	ND	ND	Crop and Ileum:<br>↑ Lactobacillus	ND	ND	ND	Crop, Ileum and Cecum:<br>↓ E.coli<br>↓ Salmonella
Yang 2008	Bacitracin	Cobb broiler chicken<br>Male<br>Healthy	Antibiotic (50 ppm)<br>E.coli (10^7 CFU/mL ;<br>1mL/animal then 10^5 CFU/mL of water)<br>3 weeks<br>Culture	=	=	=	= (jejunum)	ND	ND	ND	ND	Ileum:<br>↑ Lactobacillus	ND	=	= (Ileum)	Ileum:<br>↓ C. perfringens
		Cobb broiler chicken<br>Male<br>*E.coli*		=	=	↑ FCE	= (jejunum)	ND	ND	ND	ND	Ileum:<br>= Lactobacillus	ND	=	= (Ileum)	Ileum:<br>↓ C. perfringens
Daneshmand 2019	Bacitracin	Cobb broiler chicken<br>Male<br>*E.coli*	Antibiotic (40 mg/kg of diet)<br>E.coli (10^8 CFU)<br>3 weeks<br>Culture	↑ ADG	↑ ADFI	↓ FCR	Jejunum:<br>↑ VH<br>↑ VSA<br>= CD<br>= VH/CD ratio	Jejunum:<br>= [IL-6]<br>= [IL-2]	Jejunum:<br>= MUC2, Claudin2, Occludin mRNA	ND	ND	Ileum:<br>= Lactobacillus<br>= Bifidobacterium	ND	ND	ND	Ileum:<br>↓ E.coli
Pereira 2018	Bacitracin + Neomycin	Cobb broiler chicken<br>Male<br>Healthy	Antibiotic (40 mg/kg of diet)<br>6 weeks<br>Sequencing	=	=	=	ND	ND	ND	↓ α-diversity and richness	Cecum:<br>↑ Firmicutes<br>↓ Bacteroidetes	Small intestine:<br>↑ Lactobacillus<br>= Bifidobacterium	Small intestine:<br>↓ Bacteroides<br>= Ruminococcus<br>= Faecalibacterium	ND	ND	ND
Adeyemi 2021	Oxytetracycline + Neomycin	Ross 308 broiler chicken<br>Male and Female<br>Healthy	Antibiotic (0.4g/ kg of diet)<br>6 weeks<br>Culture	↑ BW<br>↑ BWG	↑ FI	↓ FCR	ND	Splenic:<br>↑ [IL-10]<br>↓[TNFα]<br>↓ [IgG]<br>Muscle:<br>↑ [SOD]<br>↑ [GPx]<br>↑ [Catalase]<br>↑ [TAS]	ND	ND	Cecum:<br>↑ proteobacteria	Ileum and Cecum:<br>= Lactobacillus	ND	ND	ND	Ileum and Cecum:<br>↓ E.coli<br>↓Salmonella
Wu 2021	Aureomycin	Ross 308 broiler chicken<br>Male<br>Healthy	Antibiotic (75mg/ kg of diet)<br>6 weeks<br>Sequencing	↑ BW<br>↑ ADG	=	=	Duodenum and Ileum:<br>↑ VH<br>↓ CD<br>↑ VH/CD ratio	Serum:<br>↑ [IL-1β]<br>↑ [IL-6]	ND	= α-diversity<br>Modification of β-diversity	Cecum:<br>↑ proteobacteria	ND	Cecum<br>↓ Phascolarctobacterium	ND	ND	Cecum:<br>↑ Escherichia-shigella
Xing 2016	Aureomycin	Linwu duckling<br>Female<br>Healthy	Antibiotic (150mg/ kg of diet)<br>9 weeks<br>RTqPCR	ND	ND	ND	Jejunum:<br>= VH and CD<br>↑ VH/CD ratio<br>Ileum:<br>= CD and VH/CD ratio<br>↑ VH	Jejunum:<br>↓ IL-2, IL-10, IL-18 mRNA<br>Ileum:<br>↓ IL-2, IL-8, IL-10 mRNA	ND	ND	ND	Cecum:<br>= Lactobacillus<br>= Bifidobacterium	Cecum<br>= Magasphaera elsdenii<br>= Clostridium leptum	ND	ND	Cecum:<br>= E.coli
Ruan 2021	Virginiamycin	Quingyan chicken<br>Female<br>Healthy	Antibiotic (20mg/ kg of diet)<br>4 weeks<br>Sequencing	=	=	=	ND	Jejunum:<br>↑ total antioxydant capacity	Ileum:<br>↑Claudine 1, MUC 2 and AvBD1	= α-diversity	Ileum:<br>↑ Actinobacteria<br>↓ Firmicutes	Cecum:<br>= Lactobacillus<br>= Enterococcus	ND	ND	ND	ND
Han 2013	Virginiamycin	Arbor Acres broiler chickens<br>Male and Female<br>Healthy	Antibiotic (10mg/ kg of diet)<br>3 or 6 weeks<br>RTqPCR	↑ BW<br>↑ ADG	=	↓ FCR	ND	Plasma<br>↓[MDA]<br>= [TAS] and [SOD]	ND	= α-diversity, eveness and richness	ND	ND	ND	ND	ND	ND
Johansen 2007	Salinomycin	Ross 308 broiler chicken<br>Female<br>*Camplobacter jejuni*	Antibiotic (75mg/ kg of diet)<br>Campylobacter (10^9 CFU)<br>3 or 6 weeks<br>Culture	↑ BW	ND	ND	ND	Jejunum:<br>↑ total antioxydant capacity	ND	ND	ND	Ileum and Cecum:<br>= Lactobacillus	ND	ND	ND	Ileum and Cecum:<br>= Campylobacter<br>↓ C.perfringens
Czerwinski 2012	Salinomycin	Ross 308 broiler chicken<br>Female<br>Healthy	Antibiotic (0.5g/ kg of diet)<br>4 weeks<br>FISH probing	↓ BWG	↓ FI	↑ FCE	Jejunum:<br>↓ VH<br>= CD<br>= VH/CD ratio	ND	ND	ND	ND	Ileum and Cecum:<br>↓ Lactobacillus	ND	↑ (ileum and Cecum)	Ileum:<br>↑ % acetate<br>↓[Lactic acid], [SCFA total] and % lactic acid	ND
Xia 2019	Flavomycin	Tegek broier chicken<br>Male<br>Healthy	Antibiotic (400 ppm)<br>6 weeks<br>FISH probing	↑ BW<br>↑ BWG	↓ FI	=	ND	Serum:<br>↑ [IgM]	ND	ND	ND	Cecum:<br>↑ Lactobacillus<br>↓ Bifidobacterium	Cecum:<br>= Bacteroides spp	ND	ND	ND
Xie 2020	Flavomycin	AA broiler chicken<br>Healthy	Antibiotic (62.5 mg/kg of diet)<br>6 weeks<br>FISH probing	ND	ND	ND	= VH, CD and VH/CD in duodenum, jejunum and ileum	ND	ND	ND	ND	Cecum:<br>= Lactobacillus	ND	ND	ND	Caecum:<br>= E.coli
Yang 2012	Colistine	Lignan yellow broiler chicken<br>Male<br>Healthy	Antibiotic (10 mg/kg of diet)<br>6 weeks<br>Culture	↑ BW<br>↑ ADG	ND	ND	ND	Serum:<br>↑ [IgA], [IgG], [IgM]	ND	ND	ND	Cecum:<br>↓ Lactobacillus<br>↓ Bifidobacterium	ND	ND	ND	Cecum:<br>↑ E.coli<br>↑ Salmonella<br>↑ C.perfringens
Cao 2018	Colistine	Ross 308 broiler chicken<br>Healthy	Antibiotic (20 mg/kg of diet)<br>6 weeks<br>sequencing	↑ BW<br>↑ ADG	ND	=	ND	ND	ND	ND	ND	Cecum:<br>= Lactobacillus	Cecum:<br>↓ Ruminococcus	ND	ND	ND

Table S12: Effect of antibiotics on the phenotype and gastro-intestinal microbiota of poultry (2/2)

Ref	Intervention	Conditions	Methods	Phenotype				Immunity	Intestinal barrier	Microbiota						
				Weight	Food intake	FCR or FCE	Villi structure			Diversity	Phylum	Lactic acid-producing bacteria	SCFA-producing bacteria	pH	SCFA	Pathogenic and/or Zoonotic bacteria
Feng 2020	Oxytetracycline + Nosiheptide	Yellow feather briukers<br>Healthy	Oxy (200 mg/kg of diet)<br>Nosi (250 mg/kg of diet)<br>9 weeks<br>sequencing	=	=	=	ND	ND	ND	= α-diversity, eveness and richness	= (Cecum)	Cecum:<br>= Lactobacillus<br>= Enterococcus	Cecum:<br>= Bacteroides<br>= Phascolarctobacterium<br>= Faecalibacterium	ND	ND	ND
Singh 2013	Penicillin	Cobb broiler chicken<br>Male<br>Healthy	Antibiotic (55 mg/kg of diet)<br>3 weeks<br>RTqPCR	ND	ND	ND	ND	ND	ND	↓ α-diversity	Cecum:<br>↑ Firmicutes<br>↓ Bacteroidetes	ND	Caecum<br>↑ Roseburia	ND	ND	Cecum:<br>↓ Escherichia<br>↓ Salmonella<br>↓ Campylobacter
Mountzouris 2019	Avilamycin	Cobb broiler chicken<br>Male<br>Healthy	Antibiotic (2.5 mg/kg of diet)<br>6 weeks<br>RTqPCR	ND	ND	ND	ND	Ileum and Caecum:<br>↑ [IgA]	= (Ileum and caecum)	ND	ND	Cecum:<br>↓ Lactobacillus<br>= Bifidobacterium	Ileum and Cecum:<br>= Bacteroides, clostridium leptum and coccoides	ND	= (Ileum and Caecum)	Ileum and Cecum:<br>= E.coli<br>= C. perfringens
Viveros 2011	Avoparcin	Cobb broiler chicken<br>Male<br>Healthy	Antibiotic (50 mg/kg of diet)<br>3 weeks<br>Culture	ND	ND	↓ FCR	Jejunum<br>↓ VH and CD<br>↑ VH/CD ratio	ND	ND	ND	ND	Ileum:<br>↓ Lactobacillus<br>↑ Enteroccocus<br>Cecum:<br>↑ Lactobacillus<br>↑ Enteroccocus<br>= Bifidobacterium	ND	ND	ND	Cecum:<br>↑ E.coli

Table S13: Effect of antimicrobial peptides on the phenotype and gastro-intestinal microbiota of poultry

Ref	Intervention	Conditions	Methods	Phenotype				Immunity	Intestinal barrier	Microbiota		
				Weight	Food intake	FCR or FCE	VH, CD, VH/CD ratio			Lactic acid-producing bacteria	SCFA-producing bacteria	Pathogenic and/or Zoonotic bacteria
Daneshmand 2019	cLF36	Cobb broiler chicken<br>Male<br>*E.coli*	Peptide (10 mg/kg of diet)<br>E.coli (10^8 CFU/animal)<br>3 weeks<br>Culture	↑ ADG	=	↓ FCR	Jejunum:<br>↑ VH<br>↑ VW<br>↑ VSA<br>= CD<br>= VH/CD ratio	Jejunum:<br>↓ IL-2<br>↑ IL-6	Jejunum:<br>↑ Claudin-1<br>↑ Occludin<br>↓ MUC2	Ileum:<br>↑Lactobacillus<br>= Bifidobacterium	ND	Ileum:<br>↓ E.coli

Table S14: Effect of plants and seaweed on the phenotype and gastro-intestinal microbiota of poultry (1/2)

Ref	Intervention	Conditions	Methods	Phenotype				Immunity	Microbiota		
				Weight	Food intake	FCR or FCE	Villi structure		Lactic acid-producing bacteria	SCFA-producing bacteria	Pathogenic and/or Zoonotic bacteria
Kang 2017	Chorella by products	Ross 308 broiler chicken<br>Males<br>Healthy	Plants (25, 50 or 75g/kg of diet)<br>5 weeks<br>Culture	↑ BW<br>↑ BWG	=	=	Ileum:<br>↑ VH<br>↑ CD<br>↓VH/CD ratio	Plasma<br>↑ IgG, IgM and IgA	Cecum:<br>↑ Lactobacillus	ND	Cecum:<br>↓ Salmonella<br>↓ E.coli
Ruan 2021	Oregano essential oil	Quingyan chicken<br>Female<br>Healthy	Plants (105 or 300 mg/kg of diet)<br>4 weeks<br>Sequencing	↑ BW<br>↑ BWG	↑ FI<br>↑ ADFI	=	ND	Plasma, jejunum, ileum:<br>↑[GSH-Px]<br>Jejunum:<br>↑[T-AOC]<br>↓[MDA]<br>Plasma:<br>↑[IgG]<br>↓[TGFβ]<br>Ileum:<br>↑[SIgA]<br>↓[TNFα]<br>↑[GPx1]<br>↑[SOD1]<br>↑[NRF2]	Ileum:<br>↑ Lactobacillus	ND	Ileum:<br>↑ Clostridium sensu stricto_1
Kim 2015	Capsicum / Curcuma longa	Ross 308 broiler chickens<br>Male<br>C.perfringens/E.maxima	Plants (4 mg/kg of diet)<br>C.perfringens (10^9 CFU)<br>E.maxima (10^4 oocysts)<br>3 weeks<br>Sequencing	=	ND	ND	ND	ND	Ileum:<br>↓ Lactobacillus	ND	ND
		Cobb 500 broiler chickens<br>Male<br>C.perfringens/E.maxima		↑ BW	ND	ND	ND	ND	Ileum:<br>↑ Lactobacillus	ND	ND
		Hubbard broiler chickens<br>Male<br>C.perfringens/E.maxima		↑ BW	ND	ND	ND	ND	Ileum:<br>↑ Lactobacillus	ND	ND
Taha 2019	Coriander seed powder	Arbor acres broiler chicken<br>Healthy	Plants (0.1 to 0.4% of diet)<br>6 weeks<br>Culture	↑ BW<br>↑ BWG	↑ FI	↓ FCR	ND	ND	ND	ND	Ileum:<br>↓ C.perfringens<br>↓ E.coli
Viveros 2011	Grape (pomace concentrate)	Cobb broiler chicken<br>Male<br>Healthy	Plants (60g/kg of diet)<br>3 weeks<br>Culture	=	=	↓ FCR	Jejunum:<br>= VH<br>↓ CD<br>↑ VH/CD	ND	Ileum:<br>↓ Lactobacillus<br>= Enterococcus<br>Caecum:<br>↑ Lactobacillus<br>↑ Enterococcus	ND	Ileum:<br>= E.coli<br>Caecum:<br>↑ E.coli
	Grape (Seed extract)		Plants (7.2g/kg of diet)<br>3 weeks<br>Culture	↓ BWG	=	=	Jejunum:<br>↓ VH<br>↓ CD<br>= VH/CD	ND	Ileum:<br>= Lactobacillus<br>↑ Enterococcus<br>Caecum:<br>↑ Lactobacillus<br>↑ Enterococcus	ND	Ileum:<br>= E.coli<br>Caecum:<br>↑ E.coli
Reda 2020	Red Pepper Oil	Japanese quails<br>Healthy	Plants (0.4 to 1.6g/kg of diet)<br>5 weeks<br>Culture	↑ BW<br>↑ BWG	↑	↑ FCR	ND	Plasma:<br>↓[MDA]<br>↓[IgA]<br>↑[CAT]<br>↑[GSH]	Cecum:<br>↓ Lactobacillus	ND	Cecum:<br>↓ E.coli<br>↓ Salmonella
Vase-Khavari 2018	Rhus coriaria	Ross 308 broiler chicken<br>Healthy	Plants (0.5 % of diet)<br>6 weeks<br>Culture	=	=	=	ND	ND	Cecum:<br>↑ Lactobacillus	ND	Cecum:<br>= E.coli
	Heracleum persicum			=	=	=	ND	ND	Cecum:<br>= Lactobacillus	ND	Cecum:<br>= E.coli
	Mentha piperita			=	=	=	ND	ND	Cecum:<br>↑ Lactobacillus	ND	Cecum:<br>= E.coli
Adeyemi 2021	Crescentia cujete leaf	Ross 308 broiler chicken<br>Male and Female<br>Healthy	Plants (2.5 or 5% of diet)<br>6 weeks<br>Culture	↑ BW<br>↑ BWG	↑ FI	↓ FCR	ND	ND	Ileum:<br>↑ Lactobacillus<br>Cecum:<br>= Lactobacillus	ND	Ileum and Cecum:<br>↓ E.coli<br>↓ Salmonella
	Launaea taraxacifolia leaf			↑ BW<br>↑ BWG	↑ FI	↓ FCR	ND	ND	Ileum:<br>↑ Lactobacillus<br>Cecum:<br>= Lactobacillus	ND	Ileum and Cecum:<br>↓ E.coli<br>↓ Salmonella

Table S14: Effect of plants and seaweed on the phenotype and gastro-intestinal microbiota of poultry (2/2)

Ref	Intervention	Conditions	Methods	Phenotype				Immunity	Microbiota		
				**Weight**	**Food intake**	**FCR or FCE**	**Villi structure**		**Lactic acid-producing bacteria**	**SCFA-producing bacteria**	**Pathogenic and/or Zoonotic bacteria**
Divani 2018	Plantago ovata	Ross 308 broiler chicken<br>Male<br>Healthy	Plants (5 to 20 g/kg of diet)<br>6 weeks<br>Culture	↑ BWG	=	↓ FCR	Duodenum:<br>↑ VH<br>↑ CD<br>Jejunum:<br>↑ VH<br>= CD<br>Ileum:<br>= VH<br>= CD	ND	Ileum:<br>↑ Lactobacillus	ND	Ileum:<br>↓ E.coli
Kulshreshtha 2014	Chondrus Crispus	Lohmann Brown cassic laying hens<br>Female<br>Healthy	Plants (0.5 to 2% of diet)<br>4 weeks<br>RTqPCR	↑ egg weight	=	↓ FCR	Ileum:<br>↑ VH<br>↑ CD	ND	Ileum:<br>↓ Lactobacillus acidophilus<br>↑ Bifidobacterium longum	ND	Ileum:<br>↓ C.perfringens
	Sarcodiotheca gaudichaudii			=	=	↓ FCR	Ileum:<br>↑ VH<br>= CD	ND	Ileum:<br>↑ Lactobacillus acidophilus<br>↑ Bifidobacterium longum	ND	Ileum:<br>↓ C.perfringens
Fagundes 2016	50% Corn + 50% Shorghum	Ross AP95 broiler chicken<br>Male<br>Healthy	10 days<br>Sequencing	=	=	=	Jejunum:<br>= VH<br>= CD<br>= VH/CD	ND	Small intestine and caecum:<br>= lactic acid bacteria	Small intestine:<br>= Blautia<br>= Faecalibacterium	ND
	100% Shorghum			=	=	=	Jejunum:<br>= VH<br>= CD<br>= VH/CD	ND	Small intestine:<br>= lactic acid bacteria<br>Caecum:<br>↑ Lactobacillus	Small intestine:<br>= Blautia<br>= Faecalibacterium	ND
Scocco 2016	Oregano aqueous extract	Ross 308 broiler chicken<br>Female<br>Healthy	plants (2g/kg of diet)<br>3 or 6 weeks<br>Culture	=	ND	=	ND	ND	ND	ND	Cecum:<br>↓ E.coli
Abolfathi 2019	Elecampane rhizome - methanol extracts	Ross 308 broiler chicken<br>Male<br>Healthy	plants (500 or 100 mg/kg of diet)<br>6 weeks<br>Culture	↑ BWG	=	↓ FCR	Duodenum and jejunum:<br>= VH<br>=CD<br>= VH/CD ratio<br>Ileum:<br>= VH<br>↓ CD<br>↑ VH/CD ratio	Duodenum, jejunum and Ileum:<br>↑ SOD activity<br>↑ CAT activity<br>↑ GPx activity<br>↓MDA	Ileum:<br>↑ Lactobacillus<br>= Bifidobacterium	ND	Ileum:<br>= E.coli<br>= Salmonella
	Elecampane rhizome - n-hexane extracts			↑ BWG	=	↓ FCR	Duodenum and jejunum:<br>= VH<br>=CD<br>= VH/CD ratio<br>Ileum:<br>= VH<br>↓ CD<br>= VH/CD ratio	Duodenum:<br>↑ SOD activity<br>↑ CAT activity<br>↑ GPx activity<br>↓MDA<br>Jejunum:<br>↑ CAT activity<br>↑ GPx activity<br>↓MDA	Ileum:<br>↑ Lactobacillus<br>= Bifidobacterium	ND	Ileum:<br>= E.coli<br>= Salmonella
Feng 2020	Phytosterol	Yellow-feathher briuker<br>Healthy	plants (25 mg/kg of diet)<br>9 weeks<br>Sequencing	=	=	=	ND	ND	Cecum:<br>=Lactobacillus	Cecum:<br>= Phascolaretobacterium<br>= Faecalibacterium<br>= Lachnospiraceae	ND

Table S15: Effect of minerals on the phenotype and gastro-intestinal microbiota of poultry (1/2)

Ref	Intervention	Conditions	Methods	Phenotype				Microbiota					
				**Weight**	**Food intake**	**FCR or FCE**	**Villi structure**	**Diversity**	**Phylum**	**Lactic acid-producing bacteria**	**SCFA-producing bacteria**	**SCFA**	**Pathogenic and/or Zoonotic bacteria**
Reed 2018	Zinc biofortified wheat	Cornish Cross broiler chicken<br>Male and Female<br>Healthy	Zn (46.5 µg/g of diet)<br>6 weeks<br>Sequencing	ND	ND	ND	Duodenum:<br>= villus surface area	Cecum:<br>= α diversity<br>Modification of the β-diversity	Cecum:<br>= Firmicutes<br>= Actinobacteria<br>= Proteobacteria	Cecum:<br>↑ Lactobacillus reuteri	Cecum:<br>↑ Dorea<br>↑ Lachnospiraceae	ND	ND
Shao 2014	Zinc	Cobb 500 broiler chicken<br>Male<br>Salmonella Thyphimurium	Zn (120 mg/kg of diet)<br>Salmonella (109 CFU/mL ; 0.8 mL/animal)<br>2 or 6 weeks<br>RTqPCR	↑ BW	↑ FI	=	Ileum:<br>↑ VH<br>= CD<br>↑ VH/CD ratio	Ileum:<br>= α diversity<br>= richness	ND	Ileum:<br>↑ Lactobacillus<br>= Enterococcus	ND	ND	Ileum:<br>= Salmonella Typhimurium
Wang 2014	Zinc	White king pigeons<br>Male and Female<br>Healthy	Zn (2 or 10 mg/mL ; 1 mL/animal)<br>4 weeks<br>Culture	↑ BWG	=	=	ND	ND	ND	Cecum:<br>↑ Lactobacillus<br>↓ Bifidobacterium<br>↑ Enterococcus	ND	ND	Cecum:<br>↓ E.coli
Reed 2015	Zinc	Cornish Cross broiler chicken<br>Healthy	Zn (42 µg/g of diet)<br>4 weeks<br>Sequencing	ND	ND	ND	ND	Cecum:<br>= α diversity<br>↑ chao-1 index and species number	Cecum:<br>↓ Proteobacteria<br>↓ Bacteroidetes<br>↑ Firmicutes/Proteobacteria ratio	Cecum:<br>↓ Enterococcus	ND	Cecum:<br>↑ [Acetate]<br>= [Proprionate]<br>= [Butyrate]	ND
Pineda 2012	Zinc	Ross 308 broiler chicken<br>Healthy	Zn (2 or 10 mg/kg of water)<br>7 weeks<br>Culture	=	=	=	ND	ND	ND	Cecum and Ileum:<br>= Lactic acid bacteria	ND	Ileum and Cecum:<br>= [Lactic acid]<br>= [Butyrate],<br>[Propionate] and [Acetate]	Cecum and Ileum:<br>= C. perfringens
Ptak 2015	Calcium + Phosphore	Ross 308 broiler chicken<br>Female<br>Healthy	Calcium= 0.70%<br>Phosphorus= 0.56%<br>4 weeks<br>FISH probing	=	=	=	ND	ND	ND	Ileum:<br>↓ Lactobacillus / enterococcus<br>= Streptococcus/ Lactococcus<br>= Bifidobacterium	Ileum:<br>↓ Clostridium coccoides - Eubacterium rectal<br>= Clostridium leptum	Crop and Ileum:<br>↑ [Total SCFA]<br>↑ [Acetate]<br>Crop:<br>= [Lactic acid]<br>Ileum:<br>↑ [Lactic acid]<br>Caecum:<br>= [SCFA]<br>= [Acetate]<br>↓ [Propionate]	Ileum:<br>= C. perfringens
Reed 2017	Iron biofortified carioca bean	Cornish Cross broiler chicken<br>Male and Female<br>Healthy	Fe (48.7 µg/g of diet)<br>7 weeks<br>Sequencing	ND	ND	ND	ND	Cecum:<br>= α-diversity<br>= β-diversity	Cecum:<br>↑ Proteobacteria<br>↑ Firmicutes<br>↓ Elusimicrobioa<br>↓ Euryarchaeota	Cecum:<br>↓ Enterococcus	Cecum:<br>↑ Coprococcus	ND	Cecum:<br>↑ Campylobacterales
Dias 2018	Iron biofortified carioca bean	Cornish Cross broiler chicken<br>Male and Female<br>Healthy	Fe (44.04 µg/g of diet)<br>8 weeks<br>Sequencing	↑ BW	ND	ND	Duodenum:<br>= VH	Cecum:<br>= α-diversity<br>Modification of β-diversity	Cecum:<br>= Firmicutes<br>= Proteobacteria<br>= Tenericutes<br>= Verrucomicrobia	Cecum:<br>= Streptococcus<br>= Ruminococcus	Cecum:<br>↑ Blautia	ND	ND
Beasley 2020	Iron biofortified wheat	Cornish Cross broiler chicken<br>Male and Female<br>Healthy	Fe (28.9 µg/g of diet)<br>6 weeks<br>Sequencing	=	=	=	Duodenum:<br>= VH	Cecum:<br>↓ α-diversity<br>Modification of β-diversity	Cecum:<br>↑ Actinobacteria<br>↓ Firmicutes<br>↓ Proteobacteria	Cecum:<br>↑ Bifidobacterium<br>↑ Enterococcus<br>↑ Lactobacillus	Cecum:<br>↓ Lachnospiraceae<br>↓ Coprococcus<br>↓ Ruminococcus<br>↓ Faecalibacterium	Cecum:<br>↑ [Acetic acid],<br>[Proprionic acid] and [Valeric acid]<br>↓ [Butanoic acid]	Cecum:<br>↓ Escherichia
Dias 2019	Iron biofortified carioca bean	Cornish Cross broiler chicken<br>Male and Female<br>Healthy	Fe (3.52 mg intra amniotic)<br>3 weeks	ND	ND	ND	ND	ND	ND	Cecum:<br>↓ Bifidobacterium<br>= Lactobacillus	ND	ND	Cecum:<br>↓ E.coli
			Fe (4.32 mg intra amniotic)<br>3 weeks<br>RTqPCR<br>Bean extact	ND	ND	ND	ND	ND	ND	Cecum:<br>= Bifidobacterium<br>↑ Lactobacillus	ND	ND	Cecum:<br>↑ E.coli

Table S15: Effect of minerals on the phenotype and gastro-intestinal microbiota of poultry (2/2)

Ref	Intervention	Conditions	Methods	Phenotype				Microbiota					
				Weight	Food intake	FCR or FCE	Villi structure	Diversity	Phylum	Lactic acid-producing bacteria	SCFA-producing bacteria	SCFA	Pathogenic and/or Zoonotic bacteria
Beasley 2020	Iron	Cornish Cross broiler chicken<br>Male and Female<br>Healthy	Fe (1 mg intra amniotic)<br>3 weeks<br>RTqPCR	ND	ND	ND	Duodenum:<br>= VH	ND	ND	Cecum:<br>= Bifidobacterium<br>= Lactobacillus	ND	ND	ND
			Fe-EDTA (77 µM intra amniotic)<br>3 weeks<br>RTqPCR	ND	ND	ND	Duodenum:<br>↑ VH	ND	ND	Cecum:<br>= Bifidobacterium<br>= Lactobacillus	ND	ND	ND
			Fe-Nicotinamine (1.6 mM intra amniotic)<br>3 weeks<br>RTqPCR	ND	ND	ND	Duodenum:<br>↑ VH	ND	ND	Cecum:<br>↑ Bifidobacterium<br>= Lactobacillus	ND	ND	ND
Xia 2004	Montmorillonite	Commercial broiler chicken<br>Male<br>Healthy	MMT (1.5g/kg of diet)<br>7 weeks<br>Culture	=	=	=	Jejunum:<br>↑ VH<br>= CD<br>↑ VH/CD ratio<br>Jejunum and Ileum:<br>= VH, CD and CH/CD ratio	ND	ND	Small intetine and Cecum:<br>= Lactobacillus<br>= Bifidobacterium	ND	ND	Small intetine and Cecum:<br>= E.coli
	Cooper		MMT (1.5g/kg of diet)<br>7 weeks<br>Culture	=	=	=	Duodenum, Jejunum and Ileum:<br>= VH, CD and CH/CD ratio	ND	ND	Small intetine and Cecum:<br>= Lactobacillus<br>= Bifidobacterium	ND	ND	Small intetine and Cecum:<br>= E.coli
	Montmorillonite + cooper		MMT (1.5g/kg of diet)<br>7 weeks<br>Culture	↑ BWG	=	↓ FCR	Duodenum and Ileum:<br>↑ VH<br>= CD<br>↑ VH/CD ratio<br>Jejunum:<br>↑ VH<br>↓ CD<br>↑ VH/CD ratio	ND	ND	Small intetine and Cecum:<br>= Lactobacillus<br>= Bifidobacterium	ND	ND	Small intetine and Cecum:<br>↓ E.coli

Table S16: Effect of polyphenol on the phenotype and gastro-intestinal microbiota of poultry

Ref	Intervention	Conditions	Methods	Phenotype				Immunity	Intestinal barrier	Microbiota	
				Weight	Food intake	FCR or FCE	Villi structure			Lactic acid-producing bacteria	Pathogenic and/or Zoonotic bacteria
Liu 2017	Chestnut tannins	Ross 308 broiler chicken<br>Male<br>Healthy	Tannin (1 or 2 g/kg of diet)<br>2 weeks<br>RTqPCR	↑ BWG	=	=	Jejunum:<br>↑ VH<br>= CD<br>= VH/CD ratio	Jejunum:<br>↓ [IL-6] and [NFKb]<br>↓ [MDA]<br>↑ [TAOC] and [SOD]<br>= [GSH-Px]	Jejunum:<br>↑ [ZO-1]	Jejunum and Cecum:<br>= Lactobacillus	Jejunum and Cecum:<br>↓ E.coli

Table S17: Effect of vitamins on the phenotype and gastro-intestinal microbiota of poultry

Ref	Intervention	Conditions	Methods	Phenotype				Microbiota	
				**Weight**	**Food intake**	**FCR or FCE**	**Villi structure**	**Lactic acid-producing bacteria**	**Pathogenic and/or Zoonotic bacteria**
Scocco 2016	Vitamine E	Ross 308 broiler chicken Female Healthy	Vit (150 mg/kg of diet) 3 or 6 weeks Culture	=	ND	=	ND	Ileum and Cecum: = Lactic acid bacteria	Ileum: ↑ E.coli Cecum: = E.coli

Abbreviations:

BW: Body Weight
BWG: Body Weight Gain
ADG: Average Daily Gain
FI: Feed Intake
ADFI: Average Daily Feed Intake
FCR: Feed Conversion Ratio
FCE: Feed Conversion Efficiency
VH: Villi Height
CD: Crypt Depth
= No change compared with the control group
↑ Increase compared with the control group
↓ Decrease compared with the control group
ND: Not Determined

Bibliography:

1. Abolfathi, M.-E., Tabeidian, S.A., Foroozandeh Shahraki, A.D., Tabatabaei, S.N., Habibian, M., 2019. Comparative effects of n-hexane and methanol extracts of elecampane (Inula helenium L.) rhizome on growth performance, carcass traits, feed digestibility, intestinal antioxidant status and ileal microbiota in broiler chickens. Arch. Anim. Nutr. 73, 88–110. https://doi.org/10.1080/1745039X.2019.1581027
2. Adeyemi, K.D., Audu, S., Oloke, J.A., Oladiji, O.E., Salawu, K.F., Ahmed, R.A., Sulaimon, R.O., 2021. Influence of Crescentia cujete and Launaea taraxacifolia leaves on growth, immune indices, gut microbiota, blood chemistry, carcass, and meat quality in broiler chickens. Trop. Anim. Health Prod. 53, (22 June 2021). https://doi.org/10.1007/s11250-021-02812-1
3. Atterbury, R.J., Hobley, L., Till, R., Lambert, C., Capeness, M.J., Lerner, T.R., Fenton, A.K., Barrow, P., Sockett, R.E., 2011. Effects of orally administered Bdellovibrio bacteriovorus on the well-being and Salmonella colonization of young chicks. Appl. Environ. Microbiol. 77, 5794–5806. https://doi.org/10.1128/AEM.00426-11
4. Beasley, J.T., Johnson, A.A.T., Kolba, N., Bonneau, J.P., Glahn, R.P., Ozeri, L., Koren, O., Tako, E., 2020. Nicotianamine-chelated iron positively affects iron status, intestinal morphology and microbial populations in vivo (Gallus gallus). Sci. Rep. 10, 2297. https://doi.org/10.1038/s41598-020-57598-3
5. Bin Peng, Liu ShaoJuan, Chen Shuai, Zeng ZhaoYing, Huang RuiLin, Yin YuLong, Liu Gang, 2017. The effect of aspartate supplementation on the microbial composition and innate immunity on mice. Amino Acids 49, 2045–2051. https://doi.org/10.1007/s00726-017-2467-5
6. Cao, G.T., Zhan, X.A., Zhang, L.L., Zeng, X.F., Chen, A.G., Yang, C.M., 2018. Modulation of broilers' caecal microflora and metabolites in response to a potential probiotic Bacillus amyloliquefaciens. J. Anim. Physiol. Anim. Nutr. 102, e909–e917.
7. Chen, J., Yu, Y., 2021. Bacillus subtilis-fermented products ameliorate the growth performance and alter cecal microbiota community in broilers under lipopolysaccharide challenge. Poult. Sci. 100, 875–886. https://doi.org/10.1016/j.psj.2020.10.070
8. Chen, W., Zhu, X.Z., Wang, J.P., Wang, Z.X., Huang, Y.Q., 2013. Effects of Bacillus subtilis var. natto and Saccharomyces cerevisiae fermented liquid feed on growth performance, relative organ weight,

intestinal microflora, and organ antioxidant status in Landes geese. J. Anim. Sci. 91, 978–985. https://doi.org/10.2527/jas.2012-5148

9. Czerwiński, J., Højberg, O., Smulikowska, S., Engberg, R.M., Mieczkowska, A., 2012. Effects of sodium butyrate and salinomycin upon intestinal microbiota, mucosal morphology and performance of broiler chickens. Arch. Anim. Nutr. 66, 102–116. https://doi.org/10.1080/1745039X.2012.663668
10. Daneshmand, A., Kermanshahi, H., Sekhavati, M.H., Javadmanesh, A., Ahmadian, M., 2019. Antimicrobial peptide, cLF36, affects performance and intestinal morphology, microflora, junctional proteins, and immune cells in broilers challenged with E. coli. Sci. Rep. 9. https://doi.org/10.1038/s41598-019-50511-7
11. Dias, D.M., Kolba, N., Binyamin, D., Ziv, O., Regini Nutti, M., Martino, H.S.D., Glahn, R.P., Koren, O., Tako, E., 2018. Iron Biofortified Carioca Bean (Phaseolus vulgaris L.)-Based Brazilian Diet Delivers More Absorbable Iron and Affects the Gut Microbiota In Vivo (Gallus gallus). Nutrients 10, E1970. https://doi.org/10.3390/nu10121970
12. Dias, D.M., Kolba, N., Hart, J.J., Ma, M., Sha, S.T., Lakshmanan, N., Nutti, M.R., Martino, H.S.D., Glahn, R.P., Tako, E., 2019. Soluble extracts from carioca beans (Phaseolus vulgaris L.) affect the gut microbiota and iron related brush border membrane protein expression in vivo (Gallus gallus). Food Res. Int. Ott. Ont 123, 172–180. https://doi.org/10.1016/j.foodres.2019.04.060
13. Divani, A., Bagherzadeh-Kasmani, F., Mehri, M., 2018. Plantago ovata in broiler chicken nutrition: Performance, carcass criteria, intestinal morphology, immunity, and intestinal bacterial population. J. Anim. Physiol. Anim. Nutr. 102, e353–e363. https://doi.org/10.1111/jpn.12753
14. Fagundes, N.S., Pereira, R., Bortoluzzi, C., Rafael, J.M., Napty, G.S., Barbosa, J.G.M., Sciencia, M.C.M., Menten, J.F.M., 2017. Replacing corn with sorghum in the diet alters intestinal microbiota without altering chicken performance. J. Anim. Physiol. Anim. Nutr. 101, e371–e382. https://doi.org/10.1111/jpn.12614
15. Feng, Kong FangAng, Yan Xian, Zheng LiWei, Qi QiEn, Long LiNa, Gong Li, Huang WeiLong, Zhang HuiHua, 2021. Effects of glycerol monolaurate supplementation on egg production, biochemical indices, and gut microbiota of broiler breeders at the late stage of production. Poult. Sci. 100. https://doi.org/10.1016/j.psj.2021.101386
16. Feng, X., Zhu Hui, Chen BoDong, Zhu Cui, Gong Li, Hu ZhiYong, Zhang HuiHua, 2020. Effects of phytosterols supplementation on growth performance and intestinal microflora of yellow-feather broilers. Poult. Sci. 99, 6022–6030. https://doi.org/10.1016/j.psj.2020.07.036
17. Feng Xin, Kong FangAng, Zheng LiWei, Qi QiEn, Long LiNa, Gong Li, Huang WeiLong, Zhang HuiHua, 2021. Effects of monobutyrin supplementation on egg production, biochemical indexes, and gut microbiota of broiler breeders. Poult. Sci. 100. https://doi.org/10.1016/j.psj.2020.11.074
18. Fernandez, F., Hinton, M., Gils, B. van, 2002. Dietary mannan-oligosaccharides and their effect on chicken caecal microflora in relation to Salmonella Enteritidis colonization. Avian Pathol. 31, 49–58. https://doi.org/10.1080/03079450120106000
19. Gomes, M.J.C., Martino, H.S.D., Tako, E., 2021. Effects of iron and zinc biofortified foods on gut microbiota in vivo (Gallus gallus): a systematic review. Nutrients 13. https://doi.org/10.3390/nu13010189
20. Han, W., Zhang, X.L., Wang, D.W., Li, L.Y., Liu, G.L., Li, A.K., Zhao, Y.X., 2013. Effects of microencapsulated Enterococcus fecalis CG1.0007 on growth performance, antioxidation activity, and intestinal microbiota in broiler chickens. J. Anim. Sci. 91, 4374–4382. https://doi.org/10.2527/jas.2012-5956
21. Jeong, J.S., Kim, I.H., 2014. Effect of Bacillus subtilis C-3102 spores as a probiotic feed supplement on growth performance, noxious gas emission, and intestinal microflora in broilers. Poult. Sci. 93, 3097–3103. https://doi.org/10.3382/ps.2014-04086
22. Johansen, C.H., Bjerrum, L., Pedersen, K., 2007. Impact of salinomycin on the intestinal microflora of broiler chickens. Acta Vet. Scand. 49, (26 October 2007).

23. Kang, H.K., Park, S.B., Kim, C.H., 2017. Effects of dietary supplementation with a chlorella by-product on the growth performance, immune response, intestinal microflora and intestinal mucosal morphology in broiler chickens. J. Anim. Physiol. Anim. Nutr. 101, 208–214. https://doi.org/10.1111/jpn.12566
24. Kim, J.E., Lillehoj, H.S., Hong YeongHo, Kim GeunBae, Lee, S.H., Lillehoj, E.P., Bravo, D.M., 2015. Dietary Capsicum and Curcuma longa oleoresins increase intestinal microbiome and necrotic enteritis in three commercial broiler breeds. Res. Vet. Sci. 102, 150–158. https://doi.org/10.1016/j.rvsc.2015.07.022
25. Kulshreshtha, G., Rathgeber, B., Stratton, G., Thomas, N., Evans, F., Critchley, A., Hafting, J., Balakrishnan Prithiviraj, 2014. Feed supplementation with red seaweeds, Chondrus crispus and Sarcodiotheca gaudichaudii, affects performance, egg quality, and gut microbiota of layer hens. Poult. Sci. 93, 2991–3001. https://doi.org/10.3382/ps.2014-04200
26. Liu, H.W., Li, K., Zhao, J.S., Deng, W., 2018. Effects of chestnut tannins on intestinal morphology, barrier function, pro-inflammatory cytokine expression, microflora and antioxidant capacity in heat-stressed broilers. J. Anim. Physiol. Anim. Nutr. 102, 717–726. https://doi.org/10.1111/jpn.12839
27. Markazi, A., Luoma, A., Shanmugasundaram, R., Mohnl, M., Murugesan, G.R., Selvaraj, R., 2018. Effects of drinking water synbiotic supplementation in laying hens challenged with Salmonella. Poult. Sci. 97, 3510–3518. https://doi.org/10.3382/ps/pey234
28. Mountzouris, K.C., Palamidi, I., Paraskeuas, V., Griela, E., Fegeros, K., 2019. Dietary probiotic form modulates broiler gut microbiota indices and expression of gut barrier genes including essential components for gut homeostasis. J. Anim. Physiol. Anim. Nutr. 103, 1143–1159. https://doi.org/10.1111/jpn.13112
29. Nava, G.M., Attene-Ramos, M.S., Gaskins, H.R., Richards, J.D., 2009. Molecular analysis of microbial community structure in the chicken ileum following organic acid supplementation. Vet. Microbiol. 137, 345–353. https://doi.org/10.1016/j.vetmic.2009.01.037
30. Pandi, J., Glatz, P., Forder, R., Komolong, B., Chousalkar, K., 2018. Evaluation of the effects of sweet potato (Ipomoea batatas (L.) Lam) in broiler diets. J. Anim. Physiol. Anim. Nutr. 102, e216–e224. https://doi.org/10.1111/jpn.12730
31. Peinado, M.J., Echávarri, A., Ruiz, R., Suárez-Pereira, E., Ortiz Mellet, C., García Fernández, J.M., Rubio, L.A., 2013. Effects of inulin and di-D-fructose dianhydride-enriched caramels on intestinal microbiota composition and performance of broiler chickens. Animal 7, 1779–1788. https://doi.org/10.1017/S1751731113001572
32. Pereira, R.V.V., Carroll, L.M., Lima, S., Foditsch, C., Siler, J.D., Bicalho, R.C., Warnick, L.D., 2018. Impacts of feeding preweaned calves milk containing drug residues on the functional profile of the fecal microbiota. Sci. Rep. 8, 554. https://doi.org/10.1038/s41598-017-19021-2
33. Pineda, L., Chwalibog, A., Sawosz, E., Lauridsen, C., Engberg, R., Elnif, J., Hotowy, A., Sawosz, F., Gao YuHong, Ali, A., Moghaddam, H.S., 2012. Effect of silver nanoparticles on growth performance, metabolism and microbial profile of broiler chickens. Arch. Anim. Nutr. 66, 416–429. https://doi.org/10.1080/1745039X.2012.710081
34. Ptak, A., Bedford, M.R., Świątkiewicz, S., Żyła, K., Józefiak, D., 2015. Phytase modulates ileal microbiota and enhances growth performance of the broiler chickens. PLoS ONE 10, e0119770. https://doi.org/10.1371/journal.pone.0119770
35. Rebolé, A., Ortiz, L.T., Rodríguez, M.L., Alzueta, C., Treviño, J., Velasco, S., 2010. Effects of inulin and enzyme complex, individually or in combination, on growth performance, intestinal microflora, cecal fermentation characteristics, and jejunal histomorphology in broiler chickens fed a wheat- and barley-based diet. Poult. Sci. 89, 276–286. https://doi.org/10.3382/ps.2009-00336
36. Reda, F.M., Alagawany, M., Mahmoud, H.K., Mahgoub, S.A., Elnesr, S.S., 2020. Use of red pepper oil in quail diets and its effect on performance, carcass measurements, intestinal microbiota, antioxidant indices, immunity and blood constituents. Anim. Int. J. Anim. Biosci. 14, 1025–1033. https://doi.org/10.1017/S1751731119002891

37. Reed, S., Knez, M., Uzan, A., Stangoulis, J.C.R., Glahn, R.P., Koren, O., Tako, E., 2018. Alterations in the Gut (Gallus gallus) Microbiota Following the Consumption of Zinc Biofortified Wheat (Triticum aestivum)-Based Diet. J. Agric. Food Chem. 66, 6291–6299. https://doi.org/10.1021/acs.jafc.8b01481
38. Reed, S., Neuman, H., Glahn, R.P., Koren, O., Tako, E., 2017. Characterizing the gut (Gallus gallus) microbiota following the consumption of an iron biofortified Rwandan cream seeded carioca (Phaseolus Vulgaris L.) bean-based diet. PloS One 12, e0182431. https://doi.org/10.1371/journal.pone.0182431
39. Reed, S., Neuman, H., Moscovich, S., Glahn, R.P., Koren, O., Tako, E., 2015. Chronic zinc deficiency alters chick gut microbiota composition and function. Nutrients 7, 9768–9784. https://doi.org/10.3390/nu7125497
40. Rodjan, P., Soisuwan, K., Thongprajukaew, K., Theapparat, Y., Khongthong, S., Jeenkeawpieam, J., Salaeharae, T., 2018. Effect of organic acids or probiotics alone or in combination on growth performance, nutrient digestibility, enzyme activities, intestinal morphology and gut microflora in broiler chickens. J. Anim. Physiol. Anim. Nutr. 102, e931–e940.
41. Ruan, D., Fan QiuLi, Fouad, A.M., Sun YunYun, Huang ShengShu, Wu AiJi, Lin ChuXiao, Kuang ZhiXiang, Zhang Chang, Jiang ShouQun, 2021. Effects of dietary oregano essential oil supplementation on growth performance, intestinal antioxidative capacity, immunity, and intestinal microbiota in yellow-feathered chickens. J. Anim. Sci. 99. https://doi.org/10.1093/jas/skab033
42. Ruan, D., Fouad, A.M., Fan, Q.L., Huo, X.H., Kuang, Z.X., Wang, H., Guo, C.Y., Deng, Y.F., Zhang, C., Zhang, J.H., Jiang, S.Q., 2020. Dietary L-arginine supplementation enhances growth performance, intestinal antioxidative capacity, immunity and modulates gut microbiota in yellow-feathered chickens. Poult. Sci. 99, 6935–6945. https://doi.org/10.1016/j.psj.2020.09.042
43. Scocco, P., Forte, C., Franciosini, M.P., Mercati, F., Casagrande-Proietti, P., Dall'Aglio, C., Acuti, G., Tardella, F.M., Trabalza-Marinucci, M., 2017. Gut complex carbohydrates and intestinal microflora in broiler chickens fed with oregano (Origanum vulgare L.) aqueous extract and vitamin E. J. Anim. Physiol. Anim. Nutr. 101, 676–684. https://doi.org/10.1111/jpn.12588
44. Shakouri, M.D., Iji, P.A., Mikkelsen, L.L., Cowieson, A.J., 2009. Intestinal function and gut microflora of broiler chickens as influenced by cereal grains and microbial enzyme supplementation. J. Anim. Physiol. Anim. Nutr. 93, 647–658. https://doi.org/10.1111/j.1439-0396.2008.00852.x
45. Shang, H.M., Hu, T.M., Lu, Y.J., Wu, H.X., 2010. Effects of inulin on performance, egg quality, gut microflora and serum and yolk cholesterol in laying hens. Br. Poult. Sci. 51, 791–796. https://doi.org/10.1080/00071668.2010.531005
46. Shao, Y., Lei Zhao, Yuan JianMin, Yang Ying, Guo YuMing, Zhang BingKun, 2014. Effect of zinc on growth performance, gut morphometry, and cecal microbial community in broilers challenged with Salmonella enterica serovar typhimurium. J. Microbiol. 52, 1002–1011. https://doi.org/10.1007/s12275-014-4347-y
47. Singh, P., Karimi, A., Devendra, K., Waldroup, P.W., Cho KwangKeun, Kwon, Y.M., 2013. Influence of penicillin on microbial diversity of the cecal microbiota in broiler chickens. Poult. Sci. 92, 272–276. https://doi.org/10.3382/ps.2012-02603
48. Taha, A.E., Hassan, S.S., Shewita, R.S., El-Seidy, A.A., El-Hack, M.E.A., Hussein, E.O.S., Saadeldin, I.M., Swelum, A.A., El-Edel, M.A., 2019. Effects of supplementing broiler diets with coriander seed powder on growth performance, blood haematology, ileum microflora and economic efficiency. J. Anim. Physiol. Anim. Nutr. 103, 1474–1483. https://doi.org/10.1111/jpn.13165
49. Vase-Khavari, K., Mortezavi, S.H., Rasouli, B., Khusro, A., Salem, A.Z.M., Seidavi, A., 2019. The effect of three tropical medicinal plants and superzist probiotic on growth performance, carcass characteristics, blood constitutes, immune response, and gut microflora of broiler. Trop. Anim. Health Prod. 51, 33–42. https://doi.org/10.1007/s11250-018-1656-x

50. Venardou, B., O'Doherty, J.V., Vigors, S., O'Shea, C.J., Burton, E.J., Ryan, M.T., Sweeney, T., 2021. Effects of dietary supplementation with a laminarin-rich extract on the growth performance and gastrointestinal health in broilers. Poult. Sci. 100. https://doi.org/10.1016/j.psj.2021.101179
51. Vineetha, P.G., Tomar, S., Saxena, V.K., Arun, T.S., Adil, K., 2016. Effect of laboratory isolated Lactobacillus plantarum LGFCP4 on growth performance, intestinal histomorphometry and gastrointestinal microflora population in guinea fowl. Indian J. Poult. Sci. 53, 37–43. https://doi.org/10.5958/0974-8180.2018.00015.6
52. Viveros, A., Chamorro, S., Pizarro, M., Arija, I., Centeno, C., Brenes, A., 2011. Effects of dietary polyphenol-rich grape products on intestinal microflora and gut morphology in broiler chicks. Poult. Sci. 90, 566–578. https://doi.org/10.3382/ps.2010-00889
53. Wang, X., Tian, Z., Azad, M.A.K., Zhang, W., Blachier, F., Wang, Z., Kong, X., 2021. Dietary supplementation with Bacillus mixture modifies the intestinal ecosystem of weaned piglets in an overall beneficial way. J. Appl. Microbiol. 130, 233–246. https://doi.org/10.1111/jam.14782
54. Wang, Y., Li, J., Xie, Y., Zhang, H., Jin, J., Xiong, L., Liu, H., 2021. Effects of a probiotic-fermented herbal blend on the growth performance, intestinal flora and immune function of chicks infected with Salmonella pullorum. Poult. Sci. 100. https://doi.org/10.1016/j.psj.2021.101196
55. Wang, Y., Yi, L., Zhao, M.L., Wu, J.Q., Wang, M.Y., Cheng, X.C., 2014. Effects of zinc-methionine on growth performance, intestinal flora and immune function in pigeon squabs. Br. Poult. Sci. 55, 403–408. https://doi.org/10.1080/00071668.2014.919375
56. Wu, D., Wu, S.B., Choct, M., Swick, R.A., 2017. Performance, intestinal microflora, and amino acid digestibility altered by exogenous enzymes in broilers fed wheat- or sorghum-based diets. J. Anim. Sci. 95, 740–751.
57. Wu, H., Wu, F.-T., Zhou, Q.-H., Zhao, D.-P., 2021. Comparative Analysis of Gut Microbiota in Captive and Wild Oriental White Storks: Implications for Conservation Biology. Front. Microbiol. 12. https://doi.org/10.3389/fmicb.2021.649466
58. Xia, Kong, J., Zhang GuoBing, Zhang XuXiang, Seviour, R., Kong YunHong, 2019. Effects of dietary inulin supplementation on the composition and dynamics of cecal microbiota and growth-related parameters in broiler chickens. Poult. Sci. 98, 6942–6953.
59. Xia, M.S., Hu, C.H., Xu, Z.R., 2004. Effects of copper-bearing montmorillonite on growth performance, digestive enzyme activities, and intestinal microflora and morphology of male broilers. Poult. Sci. 83, 1868–1875. https://doi.org/10.1093/ps/83.11.1868
60. Xie, J., Wang ZhongCheng, Cui Hu, Nie Hao, Zhang TieTao, Gao XiuHua, Qiao Yu, 2020. Effects of enzymatic hydrolysate of locust bean gum on digestibility, intestinal morphology and microflora of broilers. J. Anim. Physiol. Anim. Nutr. 104, 230–236. https://doi.org/10.1111/jpn.13245
61. Yang, C.M., Cao, G.T., Ferket, P.R., Liu, T.T., Zhou, L., Zhang, L., Xiao, Y.P., Chen, A.G., 2012. Effects of probiotic, Clostridium butyricum, on growth performance, immune function, and cecal microflora in broiler chickens. Poult. Sci. 91, 2121–2129. https://doi.org/10.3382/ps.2011-02131
62. Yang, W., Lee YueJia, Lu HsinYi, Chou ChungHsi, Wang ChinLing, 2019. Analysis of gut microbiota and the effect of lauric acid against necrotic enteritis in Clostridium perfringens and Eimeria side-by-side challenge model. PLoS ONE 14, e0205784. https://doi.org/10.1371/journal.pone.0205784
63. Yang, Y., Iji, P.A., Kocher, A., Mikkelsen, L.L., Choct, M., 2008. Effects of mannanoligosaccharide and fructooligosaccharide on the response of broilers to pathogenic Escherichia coli challenge. Br. Poult. Sci. 49, 550–559. https://doi.org/10.1080/00071660802290408
