# Supplementary material for: Effects of Major Families of Modulators on Performances and Gastrointestinal Microbiota of Poultry, Pigs and Ruminants: A Systematic Approach
Source: Microorganisms. 2023 May 31;11(6):1464. doi: 10.3390/microorganisms11061464 (PMC10300821; doi:10.3390/microorganisms11061464)
Supplement: Supplementary file 1 [file microorganisms-11-01464-s001.zip › Supplementary Data S2.pdf]

Supplemental Data S2: Effect of Modulators on Intestinal Microbiota and Phenotype of Pigs

Table S18: Effect of minerals on the phenotype and gastro-intestinal microbiota of pigs (1/2)

Ref	Intervention	Conditions	Methods	Phenotype						Immunity	Intestinal barrier	Microbiota			
				Weight	Food intake	FCR or FCE	Diarrhoea rate	Digestibility	Villi structure			Diversity	Lactic acid-producing bacteria	SCFA-producing bacteria	Pathogenic and/or Zoonotic bacteria
Broom 2006	Zinc	Large white x Landrace x Duroc<br>Healthy	ZnO(3100 mg/kg of diet)<br>3 weeks (post weaning)	=	=	=	=	ND	ND	Serum:<br>= [IgG]<br>Intestinal:<br>=[IgA]	ND	ND	Ileum:<br>= Lactic acid bacteria	ND	Ileum:<br>= E.coli
Wang 2018	Conventional Zinc	Topigs x Pietran<br>Male and Female<br>Healthy	ZnO (3200 mg/kg of diet)<br>1 weeks (post weaning)<br>Culture	↑ ADG	=	=	=	ND	ND	Ileum:<br>= TNFα, INFγ and IL-1β mRNA expression	Ileum:<br>= transeptiheliale electric resistance in ileum<br>= tigh junction gene expression	Ileum:<br>= Richness<br>=shanon diversity	Ileum:<br>= Lactobacillus	Ileum:<br>= Clostridium XIVa	Ileum:<br>↓ E.coli
	Alternative Zinc		ZnO (294 mg/kg of diet)<br>1 weeks (post weaning)<br>Culture	↑ ADG	=	=	=	ND	ND	Ileum:<br>= TNFα, INFγ and IL-1β mRNA expression	Ileum:<br>= transeptiheliale electric resistance in ileum<br>= tigh junction gene expression	Ileum:<br>= Richness<br>=shanon diversity	Ileum:<br>= Lactobacillus	Ileum:<br>= Clostridium XIVa	Ileum:<br>= E.coli
Vahjen 2011	Zinc oxyde	Landrace x Pietran<br>Healthy	ZnO (3042 mg/kg of diet)<br>2 weeks (post weaning)<br>Sequencing	ND	ND	ND	ND	ND	ND	ND	ND	Ileum:<br>↑ Richness<br>↑ Shannon index<br>↑ Simpson index	Ileum:<br>↓ Lactobacillus reuteri<br>↓ Lactobacillus helveticus	ND	ND
Rattingan 2020	Zinc oxyde	Boars x Large white x Landrace<br>Male and female<br>Healthy	ZnO (3100 ppm)<br>1 week (post weaning)<br>Sequencing	=	↑ ADFI	=	=	ND	ND	ND	ND	ND	ND	Colon:<br>↓ Roseburia<br>↓ Anaerostripes<br>↓ Oscillospira	Colon:<br>= Campylobacter
		Boars x Large white x Landrace<br>Male and female<br>Low crude protein	ZnO (3100 ppm)<br>1 week (post weaning)<br>Sequencing	=	=	=	↓	ND	ND	ND	ND	ND	ND	Colon:<br>↓ Roseburia	Colon:<br>= Campylobacter
Li 2021	Cooper/Zinc Montmorillonite	Duroc x Landrace x Yorkshire<br>Healthy	Zn (75 mg/kg)<br>Cu (105 mg/kg)<br>2 week (post weaning)<br>Sequencing	↑ ADG	=	↑ FCE	↓	ND	ND	ND	Jejunum:<br>= Transépithéliale resistance<br>↓ FD4 flux<br>↑ ZO-1 mRNA expression<br>↑ Claudine-1 mRNA expression<br>Colon:<br>↑ TER<br>↓ FD4 flux<br>↑ ZO-1 mRNA expression<br>↑ Claudine-1 mRNA expression	= α diversity	Colon:<br>↑ Lactococcus	ND	ND

Table S18: Effect of minerals on the phenotype and gastro-intestinal microbiota of pigs (2/2)

Ref	Intervention	Conditions	Methods	Phenotype						Immunity	Intestinal barrier	Microbiota			
				Weight	Food intake	FCR or FCE	Diarrhoea rate	Digestibility	Villi structure			Diversity	Lactic acid-producing bacteria	SCFA-producing bacteria	Pathogenic and/or Zoonotic bacteria
Kim 2021	Cooper	Duroc x Landrace x Yorkshire<br>Male and Female<br>Healthy	CuSO4 (125 ppm)<br>4 week<br>Culture	=	=	=	ND	=	Duodenum, jejunum and ileum:<br>= VH<br>= CD<br>= VH/CD ratio	Duodenum:<br>↑ Atox 1<br>↑ SOD1	ND	ND	Ileum and Cecum:<br>= Lactobacillus<br>Colon:<br>↑ Lactobacillus	ND	Ileum, cecum and colon:<br>↓ E.coli
	Copper (Hot-Melt extruded)		HME-Cu (125 ppm)<br>4 week<br>Culture	↑ ADG	=	=	ND	↑ Gross energy digestibility	Ileum:<br>= VH<br>= CD<br>= VH/CD ratio<br>Duodenum:<br>↑ VH<br>= CD<br>= VH/CD ratio<br>Jejunum:<br>↑ VH<br>↑ CD<br>↑VH/CD ratio	Duodenum:<br>↑ Atox 1	ND	ND	Ileum:<br>= Lactobacillus<br>Caecum and Colon:<br>↑ Lactobacillus	ND	Ileum, cecum and colon:<br>↓ E.coli
	Cooper chalted with methionine		Cu (125 ppm)<br>4 week<br>Culture	↑ ADG	=	=	ND	↑ Gross energy digestibility	Duodenum and Ileum:<br>= VH<br>= CD<br>= VH/CD ratio<br>Jejunum:<br>↑ VH<br>↓ CD<br>↑VH/CD ratio	Duodenum:<br>↑ Atox 1	ND	ND	Ileum, Caecum and Colon:<br>↑ Lactobacillus	ND	Ileum:<br>= E.coli<br>Cecum and colon:<br>↓ E.coli
Heyer 2019	Calcium and phosphore	Landrace x Pietran<br>Healthy	Ca (8,3g/kg of diet)<br>P (7.5 g/kg of diet)<br>Diet: Corn and soybean<br>9 week<br>Sequencing	ND	ND	ND	ND	↑ Phosphorus digestibility<br>↓ Calcium digestibility	ND	ND	ND	ND	Cecum and Colon:<br>↑ Lactobacillus<br>= Bifidobacterium	Colon:<br>↓ Clostridium cluster XIVa	ND
			Ca (8,3g/kg of diet)<br>P (7.5 g/kg of diet)<br>Diet: Corn and fiels peas<br>9 week<br>Sequencing	ND	ND	ND	ND	↓ Calcium digestibility	ND	ND	ND	ND	Cecum and Colon:<br>↑ Lactobacillus<br>= Bifidobacterium	Colon:<br>↓ Clostridium cluster XIVa	ND
Lv 2015	Sodium Selenium	Yorkshire x Landrace x Duroc<br>Healthy	Na (0.46mg/kg of diet)<br>6 week (post weaning)<br>Culture	↑ ADG	=	↓ FCR	↓	ND	ND	Blood:<br>↑ GPx acticity	ND	ND	Feces:<br>= Lactobacillus<br>= Bifidobacterium<br>= Enteroccocus	ND	Feces:<br>= E.coli

Table S19: Effect of polyphenol on the phenotype and gastro-intestinal microbiota of pigs

Ref	Intervention	Conditions	Methods	Phenotype				Microbiota				
				Weight	Food intake	FCR or FCE	Villi structure	Phylum	Lactic acid-producing bacteria	SCFA-producing bacteria	pH	SCFA
Wu 2017	Chlorogenic acid	Yorkshire x Landrace x Duroc<br>Healthy	CA (0.05% of diet)<br>9 weeks<br>Sequencing	ND	ND	ND	ND	Small intestin:<br>↑<br>Acidobacteria	Small intestin:<br>↓ Enterococcus<br>= Lactobacillus	Small intestin:<br>= Roseburia<br>= Prevotella	ND	ND
Biagi 2010	Tannins	Landrace x Pietrain<br>Male and Female<br>Healthy	Tanins (4.5 g/kg of diet)<br>4 weeks (post weaning)<br>Culture	↑ ADG	=	↓ FCR	Jejunum and Cecum:<br>= VH<br>= CD<br>Ileum:<br>= VH<br>↓ CD	ND	Jejunum and Cecum:<br>= Lactobacillus<br>Caecum:<br>= Enterococcus	ND	= (Ileum, Cecum and Jejunum)	Cecum:<br>= Total SCFA<br>= [butyrate]<br>= [acetate]<br>= [propionate]

Table S20: Effect of immunoglobulin on the phenotype and gastro-intestinal microbiota of pigs

Ref	Intervention	Conditions	Methods	Phenotype				Immunity	Intestinal barrier	Microbiota		
				Weight	Food intake	FCR or FCE	Diarrhoea rate			Lactic acid-producing bacteria	SCFA-producing bacteria	Pathogenic and/or Zoonotic bacteria
Tan 2019	IgY	Yorkshire x Landrace x Duroc<br>Healthy	Ig (500 ppm)<br>2 week (post weaning)<br>RTqPCR	=	=	=	↓	Jejunum:<br>= IL-1β, IL-6, INFγ and TNFα mRNA expression	Jejunum:<br>= Claudine-1 and Occludin-1 mRNA expression	Ileum, Cecum and Colon:<br>= Lactobacillus<br>= Bifidobacterium<br>Caecum:<br>↓ Enterococcus	Ileum, Cecum and Colon:<br>= Clostridium	Ileum:<br>↓ E.coli<br>Cecum and Colon:<br>= E.coli

Table S21: Effect of probiotics on the phenotype and gastro-intestinal microbiota of pigs (1/2)

Ref	Intervention	Conditions	Methods	Phenotype						Immunity	Microbiota				
				Weight	Food intake	FCR or FCE	Diarrhoea rate	Digestibility	Villi structure		Diversity	Phylum	Lactic acid-producing bacteria	SCFA-producing bacteria	Pathogenic and/or Zoonotic bacteria
Wang 2016	Enterococcus faecium	Large White × Landrace × Duroc<br>Healthy	Enterococcus (6.10^8 cfu/mL)<br>3 weeks (after birth)<br>Sequencing	↑ BW<br>↑ BWG	ND	ND	↓	ND	ND	ND	Cecum<br>↑ Richness	Cecum:<br>↑ Bacteroidetes<br>↓ Proteobacteria<br>↓ Actinobacteria	Cecum:<br>= Lactobacillus<br>= Enteroccocus	Cecum:<br>↑ Bacteroides<br>↑ Faecalibacterium<br>↓ Blautia	ND
Broom 2006	Enterococcus faecium	Large White × Landrace × Duroc<br>Healthy	Enterococcus ($1.4.10^9$ cfu/mL)<br>3 weeks (after weaning)<br>Sequencing	=	=	=	ND	ND	ND	Serum:<br>= [IgG]<br>Intestin:<br>= [IgA]	ND	ND	Ileum:<br>= Lactic acid bacteria	ND	Ileum:<br>= E.coli
Brousseau 2015	Pedicoccus acidilactici	Yorkshire x Landrace<br>Healthy	Probiotic ($2.5.10^9$ cfu then $3.5.10^9$ cfu per day in utero and 6.10^9 cfu per day from birth to weaning)<br>7 weeks<br>Culture	ND	ND	ND	ND	ND	ND	ND	Ileum:<br>↓ α-diversity<br>↓ Eveness<br>Modification of β-diversity	Ileum:<br>↑ Firmicutes<br>↓ Actinobacteria<br>Colon:<br>↑ Actinobacteria	Feces:<br>↑ Bifidobacterium<br>↓ Lactobacillus<br>Ileum and Colon:<br>= Bifidobacterium<br>= Lactobacillus	ND	ND
	Saccharomyces cerevisiae			ND	ND	ND	ND	ND	ND	ND	Ileum:<br>↑ α-diversity<br>↑ Eveness<br>= β-diversity	Ileum:<br>↑ Firmicutes<br>Feces:<br>↑ Actinobacteria	Feces, Ileum and Colon:<br>= Bifidobacterium<br>= Lactobacillus	ND	ND
	Pedicoccus acidilactici + Saccharomyces cerevisiae			ND	ND	ND	ND	ND	ND	ND	Ileum:<br>= α-diversity<br>= Eveness<br>Modification of β-diversity	Ileum, colon and Feces:<br>=	Feces, Ileum and Colon:<br>= Bifidobacterium<br>= Lactobacillus	ND	ND
Lan 2016	Bacillus coagulance + Bacillus lichenformis + Bacillus subtilis + Clostridium butyricum	Landrace x Yorkshire<br>Male and Female<br>Healthy	Probiotic (0.1% of diet)<br>6 weeks<br>Culture	↑ BW<br>↑ ADG	=	↓	= fecal score	↑ Dry matter, Gross energy and nitrogene digestibility	ND	Blood:<br>= % lymphocytes	ND	ND	Feces:<br>↑ Lactobacillus	ND	Feces:<br>↓ E.coli
Choi 2011	Lactobacillus acidophilus + Bacillus subtilis + Saccharomyces cerevisiae + Aspergillus oryzae	Landrace x Yorkshire x Duroc<br>Male and Female<br>Healthy	Probiotic (4.10^8 cfu/g of L. acidophilus, $4.8.10^9$ cfu/g of B. subtilis, 10^7 cfu/g of S. cerevisiae, and $4.3.10^7$ cfu/g of A. oryzae)<br>4 weeks (after birth)<br>Culture	↑ ADG	↑ ADFI	↓	ND	↑ Dry matter	ND	ND	ND	ND	Feces:<br>= Lactobacillus	ND	ND

Table S21: Effect of probiotics on the phenotype and gastro-intestinal microbiota of pigs (2/2)

Ref	Intervention	Conditions	Methods	Phenotype						Immunity	Microbiota				
				Weight	Food intake	FCR or FCE	Diarrhoea rate	Digestibility	Villi structure		Diversity	Phylum	Lactic acid-producing bacteria	SCFA-producing bacteria	Pathogenic and/or Zoonotic bacteria
Lv 2015	Lactobacillus acidophilus + Saccharomyces cerevisiae	Landrace x Yorkshire x Duroc<br>Healthy	Probiotic (3.10^{11} cfu/g of L. acidophilus and 3.10^{9} cfu/g of S. cerevisiae)<br>6 weeks (after weaning)<br>Culture	↑ ADG	=	↓	↓	ND	ND	Blood<br>= GPX activity	ND	ND	Feces:<br>↑ Lactobacillus<br>= Bifidobacterium<br>= Enterococcus	ND	Feces:<br>↓ E.coli
Wang 2019	Lactobacillus plantarum	Landrace x Yorkshire<br>Healthy	Probiotic (10^{10} CFU/2mL)<br>3 weeks (after birth)<br>Sequencing	=	ND	ND	↓	ND	Duodenum:<br>= VH<br>= CD<br>= VH/CD ratio<br>Ileum:<br>= VH<br>= CD<br>↑ VH/CD ratio<br>Jejunum:<br>= VH<br>↓CD<br>↑ VH/CD ratio	ND	Colon:<br>= α-diversity	Colon:<br>↑ Firmicutes<br>↑ Actinobacteria<br>↓ Bacteroidetes<br>↓ Fusobacteria	Colon:<br>↑ Lactobacillus	Colon:<br>↓ Fusobacterium	Colon:<br>↓ Escherichia-shigella
Shen 2009	Yeast	Large white x Landrace<br>Healthy	Probiotic (5g/kg)<br>3 weeks (after weaning)<br>Culture	=	=	=	ND	↑ Gross energy, Dry matter and crude protein digestibility	Jejunum:<br>↑ VH<br>= CD<br>↑ VH/CD ratio<br>Ileum and Duodenum:<br>= CD, VH and CD/VH ratio	Plasma:<br>= % lymphocyte CD4+ and CD8+<br>↓ [INFγ] in plasma<br>= [IL10]<br>Gut:<br>= [IL10]<br>↑ [INFγ]	ND	ND	Cecum, Colon and Rectum:<br>= Lactobacillus	ND	Cecum:<br>↓ E.coli<br>Colon and Rectum:<br>= E.coli
Hosseindoust 2016	Bacteriophage	Landrace x Yorkshire x Duroc<br>Male and Female<br>Healthy	Probiotic (1g/kg of diet)<br>5 weeks (after birth)<br>Culture	=	=	=	↓	= Digestibility	Jejunum:<br>↑ VH<br>= CD<br>= VH/CD ratio<br>Duodenum and Ileum:<br>= VH<br>= CD<br>= VH/CD ratio	Serum:<br>= [Ig]	ND	ND	Cecum:<br>= Lactobacillus<br>↑ Bifidobacterium<br>Ileum:<br>↑ Lactobacillus<br>= Bifidobacterium	ND	ND
		Landrace x Yorkshire x Duroc<br>Male and Female<br>Salmonella typhimurium, enteritidis and cholerasuis, E.coli and C.perfringens	Probiotic (1g/kg of diet)<br>5 weeks (after birth)<br>Culture<br>Infection: ND	=	=	↓	↓	= Digestibility	Jejunum:<br>↑ VH<br>= CD<br>= VH/CD ratio<br>Duodenum and Ileum:<br>= VH<br>= CD<br>= VH/CD ratio	Serum:<br>= [Ig]	ND	ND	Cecum:<br>= Lactobacillus<br>↑ Bifidobacterium<br>Ileum:<br>↑ Lactobacillus<br>= Bifidobacterium	ND	ND

Table S22: Effect of probiotics combined with other interventions on the phenotype and gastro-intestinal microbiota of pigs

Ref	Intervention	Conditions	Methods	Phenotype				Immunity	Microbiota			
				Weight	Food intake	FCR or FCE	Diarrhoea rate		Diversity	Lactic acid-producing bacteria	SCFA-producing bacteria	Pathogenic and/or Zoonotic bacteria
Lv 2015	*Lactobacillus acidophilus* + *Sacchaomyces cerevisiae* + Na-Selenium	Yorkshire x Landrace x Duroc<br>Healthy	Na (0.46mg/kg of diet)<br>Probiotic (3.1011 cfu/g of L. acidophilus and 3.109 cfu/g of S. cerevisiae)<br>6 week (post weaning)<br>Culture	↑BW<br>↑ ADG	=	↓FCR	↓	Blood:<br>↑ GPx acticity	ND	Feces:<br>↑ Lactobacillus<br>= Bifidobacterium<br>= Enteroccocus	ND	Feces:<br>↓ E.coli
Lo verso 2020	Cranberry + Carvacrol + Yeast + Vitamins A,D,E and B complex	Yorkshire x Landrace x Duroc<br>Healthy	Cranberry (1g/kg of diet)<br>Carvacrol (0.1 g/kg of diet)<br>Yeast (5g/kg of diet)<br>Vitamins complex (3g/kg of diet)<br>6 weeks (post weaning)<br>Sequencing	=	=	=	ND	ND	= α diversity<br>= β diversity	Colon:<br>↓ Lactobacillus amylovorus	Colon:<br>= Faecalibacterium prausnitzii	ND

Table S23: Effect of postbiotics on the phenotype and gastro-intestinal microbiota of pigs

Ref	Intervention	Conditions	Methods	Phenotype					Microbiota				
				**Weight**	**Food intake**	**FCR or FCE**	**Digestibility**	**Villi structure**	**Lactic acid-producing bacteria**	**SCFA-producing bacteria**	**pH**	**SCFA**	**Pathogenic and/or Zoonotic bacteria**
Biagi 2007	Sodium Butyrate	German Landeace x Pietrain<br>Healthy	Postbiotic (4000 ppm)<br>4 weeks (post weaning)<br>Culture	=	=	=	ND	Jejunum, Ileum and Cecum:<br>= VH<br>= CD<br>= VH/CD ratio	ND	ND	ND	Jejunum, Ileum and Cecum:<br>= [Acetate], [Propionate], [Butyrate], [Lactate]	ND
Hanczakowska 2013	Propionic acid + Formic acid	Landrace x Large white x Duroc x Pietrain<br>Healthy	Postbiotic (0.5% of diet)<br>1 weeks (post weaning)<br>Culture	=	ND	=	=	Ileum:<br>= VH<br>↑ CD<br>= VH/CD ratio	ND	ND	= Stomach, Duodenum, Jejunum, Ileum and Cecum	Ileum:<br>↑ [Valerate]<br>= [Acetate]<br>= [Proprionate]<br>= [Butyrate]<br>Cecum:<br>↑ [Isobutyrate]<br>= [Acetate]<br>= [Proprionate]<br>= [Butyrate]	Ileum:<br>= E.coli<br>= C. perfringens
	Caprylic acid + Propionic acid and Formic acid		Caprylic acid (0.2% of diet)<br>Propionic of formic acid (0.5% of diet)<br>11 week<br>Culture	↑ BW<br>↑ BWG	ND	=	↑ Crude fiber digestibility	Ileum:<br>= VH<br>= VH/CD ratio<br>↑VW<br>↑ CD	ND	ND	↑ (stomach)	Cecum:<br>↑ [butyric]<br>↑ [valeric]	Ileum:<br>= E.coli<br>= C.perfringens<br>= Candida albicans
	Capric acid + Propionic acid and Formic acid			↑ BW<br>↑ BWG	ND	=	↑ Crude fiber, protein and fat digestibility	Ileum:<br>↑ VH<br>= VH/CD ratio<br>↑VW<br>↑ CD	ND	ND	↑ (stomach)<br>↓ (jejunum)	Cecum:<br>↑ [butyric]	Ileum:<br>= E.coli<br>= C.perfringens<br>= Candida albicans
	Caprylic + Capric acid + Propionic acid and Formic acid			↑ BW<br>↑ BWG	ND	=	↑ Crude fat digestibility	Ileum:<br>= VH<br>= VH/CD ratio<br>↑VW<br>= CD	ND	ND	↑ (stomach)	Cecum:<br>= [SCFA]	Ileum:<br>= E.coli<br>= C.perfringens<br>= Candida albicans

Table S24: Effect of infection on the phenotype and gastro-intestinal microbiota of pigs

Ref	Infection	Conditions	Methods	Phenotype						Immunity	Microbiota	
				**Weight**	**Food intake**	**FCR or FCE**	**Diarrhoea rate**	**Digestibility**	**Villi structure**		**Lactic acid-producing bacteria**	**Pathogenic and/or Zoonotic bacteria**
Hosseindoust 2016	*Salmonella typhimurium, enteritidis and cholerasuis* + *E.coli* + *C.perfringens*	Landrace x Yorkshire x Duroc<br>Male and Female	Infection (ND)<br>5 weeks (after birth)<br>Culture	↓ ADG	↓ADFI	=	↑	=	Duodenum, Jejunum and Ileum:<br>= VH<br>= CD<br>= VH/CD ratio	Serum:<br>↑ [IgA]	Cecum:<br>= Lactobacillus<br>↓ Bifidobacterium<br>Ileum:<br>↓ Lactobacillus<br>= Bifidobacterium	ND

Table S25: Effect of prebiotics on the phenotype and gastro-intestinal microbiota of pigs

Ref	Intervention	Conditions	Methods	Phenotype					Immunity	Microbiota						
				Weight	Food intake	FCR or FCE	Diarrhoea rate	Villi structure		Diversity	Phylum	Lactic acid-producing bacteria	SCFA-producing bacteria	pH	SCFA	Pathogenic and/or Zoonotic bacteria
Li 2018	Inulin	Pietran x Landrace<br>Male and Female<br>Healthy	Prebiotic (0.4 to 3 g/animal/day)<br>7 week (post birth)<br>RTqPCR	↑ BW	ND	ND	ND	Jejunum:<br>↑ VH<br>= CD<br>= VH/CD<br>Ileum:<br>↑ VH<br>↑ CD<br>↓ VH/CD ratio	Colon:<br>↓ IL8, TNFα and TLR4 mRNA expression	ND	Colon:<br>= Firmicutes<br>= Bacteroidetes	Colon:<br>= Lactobacillus<br>= Bifidobacterium	Colon:<br>= Clostridium XIVa	ND	Cecum:<br>= Total SCFA<br>Colon:<br>↓ Total SCFA	Colon:<br>= E.coli
Tian 2019	GOS	Duroc x Landrace x Large white<br>Healthy	Prebiotic (1g/kg of BW)<br>3 week (post birth)<br>Sequencing	ND	ND	ND	ND	ND	Ileum<br>↑ IL10 and PBD-A mRNA expression<br>= IL8 mRNA expression	= α-diversity<br>Modification of the β-diversity	Ileum:<br>↑ Firmicute<br>↓ Protéobacteria<br>↓ Bacteroidetes	Ileum:<br>↑ Lactobacillus	ND	= (Ileum)	Ileum:<br>↑ [Butyrate]<br>= [Lactacte]	Ileum:<br>↓ Escherichia<br>↓ Clostridium sensu stricto
Rattingan 2020	Laminarin	Boars x Landrace x Large white<br>Male and Female<br>Healthy	Prebiotic (300ppm)<br>Protein (21%)<br>1 week (post weaning)<br>Sequencing	=	=	=	=	ND	ND	= α-diversity<br>= Richness	Colon:<br>= Firmicute<br>= Protéobacteria	ND	Colon:<br>= Roseburia<br>= Bacteroides	ND	Cecum and Colon:<br>= Total SCFA	Colon:<br>= Campylobacter
		Boars x Landrace x Large white<br>Male and Female<br>Reducing crude protein	Prebiotic (300ppm)<br>Protein (18%)<br>1 week (post weaning)<br>Sequencing	=	↑	=	↓	ND	ND	= α diversity<br>= Richness	Colon:<br>= Firmicute<br>= Protéobacteria	ND	Colon:<br>= Roseburia<br>= Bacteroides	ND	Cecum and Colon:<br>= Total SCFA	Colon:<br>= Campylobacter

Table S26: Effect of fibres on the phenotype and gastro-intestinal microbiota of pigs

Ref	Intervention	Conditions	Methods	Phenotype			Immunity	Microbiota					
				Weight	Food intake	FCR or FCE		Diversity	Phylum	Lactic acid-producing bacteria	SCFA-producing bacteria	SCFA	Pathogenic and/or Zoonotic bacteria
Wu 2020	50% guar gum<br>50% cellulose	Yorkshire x Landrace<br>Female<br>Pregnant	Prebiotic (3% of feed)<br>17 week<br>Sequencing	ND	ND	ND	Serum:<br>↓ [INFγ]<br>↑ [IL-10]	↑α diversity<br>Modification of the β diversity	Feces:<br>↑ Bacteroidetes	Feces:<br>= Enterococcus<br>= Lactococcus	Feces:<br>↑ Roseburia<br>↑ Bacteroides<br>= Faecalibacterium<br>= Akkermansia	Feces:<br>↑ [Butyrate]<br>↑<br>[Propionate]	ND
Yan 2016	Amylose	Yorkshire x Landrace x Duroc<br>Female<br>Pregnant	Prebiotic (54,5/100g of diet)<br>11 week<br>RTqPCR	ND	ND	ND	ND	↑ richness	ND	Feces:<br>= Bifidobacterium	Feces:<br>= Bacteroidetes	ND	ND

Table S27: Effect of amino acids on the phenotype and gastro-intestinal microbiota of pigs

Ref	Intervention	Conditions	Methods	Phenotype			Immunity	Intestinal barrier	Microbiota				
				Weight	Food intake	FCR or FCE			Diversity	Phylum	Lactic acid-producing bacteria	SCFA-producing bacteria	Pathogenic and/or Zoonotic bacteria
Chang 2021	5-Aminolevulinic acid	Landrace x Large white<br>Female<br>Healthy	AA (50 mg/kg of diet)<br>4 weeks<br>Sequencing	ND	ND	ND	ND	ND	= α diversity<br>Modification of the β diversity	Feces:<br>= phylum abundance	ND	ND	ND
Xu 2014	N acetyl cysteine	Landrace x Duroc<br>Healthy	AA (500 mg/kg of diet)<br>3 weeks (Post birth)<br>RTqPCR	ND	ND	ND	Colon:<br>↑ [T-AOC], [GPx]<br>↓ [MDA] and [NO]<br>Caecum:<br>↑ [T-AOC], [SOD]	ND	ND	ND	Ileum and Colon:<br>↑ Lactobacillus<br>Ileum, Cecum and Colon:<br>↑ Bifidobacterium	ND	Ileum and Cecum:<br>↓ E.coli
Li 2019	D Aspartate	Landrace x Duroc x Yorkshire<br>Healthy	AA (1%of diet)<br>5 weeks (Post weaning)<br>Sequencing	↓ ADG	=	=	Blood:<br>↓[IgM]	Ileum:<br>↓ TLR4 and NOD1 mRNA expression	= Evenesse, Richnesse and α diversity	Ileum:<br>= Phylum abundance	Ileum<br>= Lactobacillus<br>= Pediococcus	ND	Ileum:<br>= Escherichia shigella<br>↑ Clostridium sensu stricto 1
	L Aspartate			=	=	=	Blood:<br>= [IgM]	Ileum:<br>↓ NOD1 mRNA expression	↑ Shanon and simpson indice	Ileum:<br>↑ Chloroflexi,<br>↑ Nitrospirae<br>↑ Germatimonadetes<br>↑ Acidobacteria<br>↑ Chlorobi	Ileum<br>↓ Lactobacillus<br>↓ Pediococcus	ND	Ileum:<br>↓ Escherichia shigella<br>= Clostridium sensu stricto 1
	D and L Aspartate			=	=	=	Blood:<br>↑ [IgM]	Ileum:<br>↓ TLR4 and NOD1 mRNA expression	↑ Simpson index	Ileum:<br>↓ Protéobacteria	Ileum<br>= Lactobacillus<br>= Pediococcus	ND	Ileum:<br>= Clostridium sensu stricto 1<br>↓ Escherichia shigella

Table S28: Effect of proteins on the phenotype and gastro-intestinal microbiota of pigs

Ref	Intervention	Conditions	Methods	Phenotype				Microbiota					
				Weight	Food intake	FCR or FCE	Diarrhoea rate	Diversity	Phylum	Lactic acid-producing bacteria	SCFA-producing bacteria	SCFA	Pathogenic and/or Zoonotic bacteria
Rattingan 2020	Protein	Boars x Landrace x Large white<br>Male and Female<br>Healthy	Protein (21%)<br>1 week (post weaning)<br>Sequencing	=	=	=	=	= α diversity<br>= Richness	Colon:<br>= Firmicute<br>↑ Protéobacteria	ND	Colon:<br>= Roseburia<br>= Bacteroides	Cecum and Colon:<br>= Total SCFA	Colon:<br>= Campylobacter

Table S29: Effect of antibiotics on the phenotype and gastro-intestinal microbiota of pigs

Intervention	Conditions	Methods	Phenotype					Immunity	Microbiota				
			Weight	Food intake	FCR or FCE	Diarrhoea rate	Villi structure		Diversity	Phylum	Lactic acid-producing bacteria	SCFA-producing bacteria	Pathogenic and/or Zoonotic bacteria
Chlortetracycline	Landrace x Yorkshire x Duroc<br>Male and Female<br>Healthy	Antibiotic (0.10% of diet)<br>4 weeks (after birth)<br>Culture	↑ ADG	↑ ADFI	↓ FCR	ND	ND	ND	ND	ND	Feces:<br>= Lactobacillus	ND	ND
Chlortetracycline	Landrace x Large white<br>Healthy	Antibiotic (80 mg/kg of diet)<br>3 weeks (post weaning)<br>Culture	↑ ADG	=	=	ND	Jejunum:<br>↑ VH<br>=CD<br>↑ VH/CD ratio<br>Ileum<br>Duodenum:<br>=VH<br>=CD<br>= VH/CD ratio	Plasma:<br>= % lymphocyte CD4+ and CD8+<br>↓ [INFγ]<br>= [IL10]	ND	ND	Cecum, Colon, Rectum:<br>= Lactobacillus	ND	Cecum:<br>↓ E.coli
Chlortetracycline	Yorkshire x Landrace x Duroc<br>Healthy	Antibiotic (1.5g/kg of diet)<br>6 weeks (post weaning)<br>Sequencing	=	ND	ND	ND	ND	ND	= Richness, evenness and shanon index in Ileum and colon<br>= β diversity	ND	Ileum:<br>↓ Lactobacillus reuteri<br>Colon:<br>↓ Lactobacillus amylovorus	ND	ND
Chlortetracycline + Tiamulin	Yorkshire x Landrace<br>Healthy	Antibiotic (100mg/kg of diet)<br>7 weeks<br>Culture	ND	ND	ND	ND	ND	ND	↓ Eveness index<br>↓ α diversity<br>Modification of the β diversity	Ileum:<br>↑ Firmicutes<br>Colon:<br>↑ Actinobacteria<br>Feces:<br>↓ Actinobacteria	Feces:<br>↑ Lactobacillus	ND	ND
Colistin sulphate + Enramycin	Yorkshire x Landrace x Duroc<br>Healthy	Colistin (100 ppm)<br>Enramycin (15ppm)<br>2 week (post weaning)<br>RTqPCR	=	=	=	=	ND	Jejunum:<br>= inflammation protein expression	ND	ND	Ileum, cecum and colon:<br>↓ Lactobacillus<br>= Enteroccocus<br>= Bifidobacterium	ND	Ileum, cecum:<br>↓ E.coli
Tylosin phosphate	Topics hybrid x Pietran<br>Healthy	Antibiotic (100mg/kg of diet)<br>6 weeks (post weaning)<br>Sequencing	↑ BW	ND	↓ FCR	ND	ND	ND	= α diversity	Cecum:<br>↑ Firmicutes<br>↓ Tenericutes	ND	Colon:<br>↑ Coprococcus<br>↑ Blautia<br>↑ Ruminococcus<br>Cecum:<br>↑ Phascolarctobacterium<br>↓ Prevotella	Cecum:<br>↓ Campylobacter
Tilmicosin		Antibiotic (400 mg/kg of diet)<br>3 weeks (post weaning)<br>Culture	↑ BWG	↑ ADFI	=	ND	Jejunum:<br>= VH<br>= CD<br>= VH/CD ratio	Blood:<br>= [IgA]<br>↑ [IgM]	= shanon diversity index	ND	Feces:<br>= Lactobacillus	ND	ND
Amoxicillin	Landrace x Large white<br>Male and Female<br>Healthy	Antibiotic (600 mg/kg of diet)<br>3 weeks (post weaning)<br>Culture	↑ BWG	↑ ADFI	=	ND	Jejunum:<br>= VH<br>= CD<br>= VH/CD ratio	Blood:<br>= [IgA]<br>↑ [IgM]	= shanon diversity index	ND	Feces:<br>= Lactobacillus	ND	ND
Doxycycline		Antibiotic (300 mg/kg of diet)<br>3 weeks (post weaning)<br>Culture	↑ BWG	↑ ADFI	=	ND	Jejunum:<br>= VH<br>= CD<br>= VH/CD ratio	Blood:<br>= [IgA]<br>↑ [IgM]	= shanon diversity index	ND	Feces:<br>= Lactobacillus	ND	ND

Table S30: Effect of plants and seaweed on the phenotype and gastro-intestinal microbiota of pigs

Ref	Intervention	Conditions	Methods	Phenotype					Microbiota					
				Weight	Food intake	FCR or FCE	Digestibility	Villi structure	Diversity	Lactic acid-producing bacteria	SCFA-producing bacteria	pH	SCFA	Pathogenic and/or Zoonotic bacteria
Chen 2018	Sangrovit ® Macleata cordata	Landrace x Large white x Duroc Male and Female Healthy	Plants (50mg/kg of diet) 3 weeks (post weaning) RTqPCR	↑ ADG	↑	=	ND	Duodenum: ↓ VH = CD = VH/CD ratio Jejunum: = VH ↓ CD ↑ VH/CD ratio Ileum: ↑ VH = CD ↑ VH/CD ratio	ND	Ileum and Cecum: ↑ Lactobacillus	ND	ND	Ileum and Cecum: ↑ [Acetate] ↑ [Propionate] ↑ [Butyrate] ↑ [total SCFA]	Ileum and Cecum: ↓ Salmonella ↓ E.coli
Ivarsson 2012	Chicory forage	Yorkshire Male and Female Healthy	Plants (80g/kg of diet) 3 weeks (post birth) Culture	=	=	=	↑ Non starch polysaccharide, arabinose, xylose, uronic acid and total dietary fiber digestibility	ND	= simpson index	Feces: ↓ Lactobacillus	Feces: ↑ Prevotella ↓ Megasphera	= (feces)	ND	ND
	Chicory root			=	=	=	↑ arabinose, uronic acid and total dietary fiber digestibility	ND	= simpson index	Feces: = Lactobacillus	Feces: = Prevotella = Megasphera	↓ (feces)	ND	ND
	Chicory forage + root			=	=	=	↑ Non starch polysaccharide, arabinose, uronic acid and total dietary fiber digestibility	ND	= simpson index	Feces: ↑ Lactobacillus johnsonni	Feces: = Prevotella ↑ Megasphera	= (feces)	ND	ND

<u>Abbreviations:</u>
BW: Body Weight
BWG: Body Weight Gain
ADG: Average Daily Gain
FI: Feed Intake
ADFI: Average Daily Feed Intake
FCR: Feed Conversion Ratio
FCE: Feed Conversion Efficiency
VH: Villi Height
CD: Crypt Depth
= No change compared with the control group
↑ Increase compared with the control group
↓ Decrease compared with the control group
ND: Not Determined

Bibliography

1. Biagi, G., Piva, A., Moschini, M., Vezzali, E., Roth, F.X., 2007. Performance, intestinal microflora, and wall morphology of weanling pigs fed sodium butyrate. J. Anim. Sci. 85, 1184–1191. https://doi.org/10.2527/jas.2006-378
2. Biagia, G., Cipollini, I., Paulicks, B.R., Roth, F.X., 2010. Effect of tannins on growth performance and intestinal ecosystem in weaned piglets. Arch. Anim. Nutr. 64, 121–135. https://doi.org/10.1080/17450390903461584
3. Bosi, P., Merialdi, G., Scandurra, S., Messori, S., Bardasi, L., Nisi, I., Russo, D., Casini, L., Trevisi, P., 2011. Feed supplemented with 3 different antibiotics improved food intake and decreased the activation of the humoral immune response in healthy weaned pigs but had differing effects on intestinal microbiota. J. Anim. Sci. 89, 4043–4053. https://doi.org/10.2527/jas.2010-3311
4. Broom, L.J., Miller, H.M., Kerr, K.G., Knapp, J.S., 2006. Effects of zinc oxide and Enterococcus faecium SF68 dietary supplementation on the performance, intestinal microbiota and immune status of weaned piglets. Res. Vet. Sci. 80, 45–54. https://doi.org/10.1016/j.rvsc.2005.04.004
5. Brousseau, J.-P., Talbot, G., Beaudoin, F., Lauzon, K., Roy, D., Lessard, M., 2015. Effects of probiotics Pediococcus acidilactici strain MA18/5M and Saccharomyces cerevisiae subsp. boulardii strain SB-CNCM I-1079 on fecal and intestinal microbiota of nursing and weanling piglets. J. Anim. Sci. 93, 5313–5326. https://doi.org/10.2527/jas.2015-9190
6. Chang, M., Li, Mingcong, Li, Min, Xie, Y., Li, Y., Yang, W., Gao, Z., 2021. Changes of gut microbiota in pregnant sows induced by 5-Aminolevulinic acid. Res. Vet. Sci. 136, 57–65. https://doi.org/10.1016/j.rvsc.2021.01.021
7. Chen, J., Kang, B., Zhao, Y., Yao, K., Fu, C., 2018. Effects of natural dietary supplementation with Macleaya cordata extract containing sanguinarine on growth performance and gut health of early-weaned piglets. J. Anim. Physiol. Anim. Nutr. 102, 1666–1674. https://doi.org/10.1111/jpn.12976
8. Choi, J.Y., Kim, J.S., Ingale, S.L., Kim, K.H., Shinde, P.L., Kwon, I.K., Chae, B.J., 2011. Effect of potential multimicrobe probiotic product processed by high drying temperature and antibiotic on performance of weanling pigs. J. Anim. Sci. 89, 1795–1804. https://doi.org/10.2527/jas.2009-2794
9. Hanczakowska, E., Szewczyk, A., Swiatkiewicz, M., Okoń, K., 2013. Short- and medium-chain fatty acids as a feed supplement for weaning and nursery pigs. Pol. J. Vet. Sci. 16, 647–654. https://doi.org/10.2478/pjvs-2013-0092
10. Heyer, C.M.E., Schmucker, S., Burbach, K., Weiss, E., Eklund, M., Aumiller, T., Capezzone, F., Steuber, J., Rodehutscord, M., Hoelzle, L.E., Seifert, J., Mosenthin, R., Stefanski, V., 2019. Phytate degradation, intestinal microbiota, microbial metabolites and immune values are changed in growing pigs fed diets with varying calcium-phosphorus concentration and fermentable substrates. J. Anim. Physiol. Anim. Nutr. 103, 1185–1197. https://doi.org/10.1111/jpn.13088
11. Hosseindoust, A.R., Lee, S.H., Kim, J.S., Choi, Y.H., Kwon, I.K., Chae, B.J., 2017. Productive performance of weanling piglets was improved by administration of a mixture of bacteriophages, targeted to control Coliforms and Clostridium spp. shedding in a challenging environment. J. Anim. Physiol. Anim. Nutr. 101, e98–e107. https://doi.org/10.1111/jpn.12567
12. Ivarsson, E., Liu, H.Y., Dicksved, J., Roos, S., Lindberg, J.E., 2012. Impact of chicory inclusion in a cereal-based diet on digestibility, organ size and faecal microbiota in growing pigs. Anim. Int. J. Anim. Biosci. 6, 1077–1085. https://doi.org/10.1017/S1751731111002709
13. Kim, M., Hosseindoust, A., Choi, Y., Lee, J., Kim, K., Kim, T., Cho, H., Kang, W., Chae, B., 2021. Effects of Hot-Melt Extruded Nano-Copper as an Alternative for the Pharmacological Dose of Copper Sulfate in Weanling Pigs. Biol. Trace Elem. Res. 199, 2925–2935. https://doi.org/10.1007/s12011-020-02426-y
14. Lan, R.X., Lee, S.I., Kim, I.H., 2016. Effects of multistrain probiotics on growth performance, nutrient digestibility, blood profiles, faecal microbial shedding, faecal score and noxious gas emission in weaning pigs. J. Anim. Physiol. Anim. Nutr. 100, 1130–1138. https://doi.org/10.1111/jpn.12501

15. Li, B., Schroyen, M., Leblois, J., Wavreille, J., Soyeurt, H., Bindelle, J., Everaert, N., 2018. Effects of inulin supplementation to piglets in the suckling period on growth performance, postileal microbial and immunological traits in the suckling period and three weeks after weaning. Arch. Anim. Nutr. 72, 425–442. https://doi.org/10.1080/1745039X.2018.1508975
16. Li, X., Wen, J., Jiao, L., Wang, C., Hong, Q., Feng, J., Hu, C., 2021. Dietary copper/zinc-loaded montmorillonite improved growth performance and intestinal barrier and changed gut microbiota in weaned piglets. J. Anim. Physiol. Anim. Nutr. 105, 678–686. https://doi.org/10.1111/jpn.13522
17. Li, Y., Han, H., Yin, J., He, X., Tang, Z., Li, T., Yao, K., Yin, Y., 2019. d- and l-Aspartate regulates growth performance, inflammation and intestinal microbial community in young pigs. Food Funct. 10, 1028–1037. https://doi.org/10.1039/c8fo01410h
18. Lo Verso, L., Talbot, G., Morissette, B., Guay, F., Matte, J.J., Farmer, C., Gong, J., Wang, Q., Bissonnette, N., Beaulieu, C., Lessard, M., 2020. The combination of nutraceuticals and functional feeds as additives modulates gut microbiota and blood markers associated with immune response and health in weanling piglets. J. Anim. Sci. 98, skaa208. https://doi.org/10.1093/jas/skaa208
19. Lv, C.H., Wang, T., Regmi, N., Chen, X., Huang, K., Liao, S.F., 2015. Effects of dietary supplementation of selenium-enriched probiotics on production performance and intestinal microbiota of weanling piglets raised under high ambient temperature. J. Anim. Physiol. Anim. Nutr. 99, 1161–1171. https://doi.org/10.1111/jpn.12326
20. Rattigan, R., Sweeney, T., Vigors, S., Rajauria, G., O'Doherty, J.V., 2020. Effects of reducing dietary crude protein concentration and supplementation with laminarin or zinc oxide on the faecal scores and colonic microbiota in newly weaned pigs. J. Anim. Physiol. Anim. Nutr. 104, 1471–1483. https://doi.org/10.1111/jpn.13428
21. Shen, Y.B., Piao, X.S., Kim, S.W., Wang, L., Liu, P., Yoon, I., Zhen, Y.G., 2009. Effects of yeast culture supplementation on growth performance, intestinal health, and immune response of nursery pigs. J. Anim. Sci. 87, 2614–2624. https://doi.org/10.2527/jas.2008-1512
22. Tan, X., Li, J., Li, Y., Li, J., Wang, Q., Fang, L., Ding, X., Huang, P., Yang, H., Yin, Y., 2019. Effect of chicken egg yolk immunoglobulins on serum biochemical profiles and intestinal bacterial populations in early-weaned piglets. J. Anim. Physiol. Anim. Nutr. 103, 1503–1511. https://doi.org/10.1111/jpn.13129
23. Tian, S., Wang, Jue, Yu, H., Wang, Jing, Zhu, W., 2019. Changes in Ileal Microbial Composition and Microbial Metabolism by an Early-Life Galacto-Oligosaccharides Intervention in a Neonatal Porcine Model. Nutrients 11, E1753. https://doi.org/10.3390/nu11081753
24. Vahjen, W., Pieper, R., Zentek, J., 2011. Increased dietary zinc oxide changes the bacterial core and enterobacterial composition in the ileum of piglets. J. Anim. Sci. 89, 2430–2439. https://doi.org/10.2527/jas.2010-3270
25. Wang, Q., Sun, Q., Qi, R., Wang, J., Qiu, X., Liu, Z., Huang, J., 2019. Effects of Lactobacillus plantarum on the intestinal morphology, intestinal barrier function and microbiota composition of suckling piglets. J. Anim. Physiol. Anim. Nutr. 103, 1908–1918. https://doi.org/10.1111/jpn.13198
26. Wang, W., Van Noten, N., Degroote, J., Romeo, A., Vermeir, P., Michiels, J., 2019. Effect of zinc oxide sources and dosages on gut microbiota and integrity of weaned piglets. J. Anim. Physiol. Anim. Nutr. 103, 231–241. https://doi.org/10.1111/jpn.12999
27. Wang, Y.B., Du, W., Fu, A.K., Zhang, X.P., Huang, Y., Lee, K.H., Yu, K., Li, W.F., Li, Y.L., 2016. Intestinal microbiota and oral administration of Enterococcus faecium associated with the growth performance of new-born piglets. Benef. Microbes 7, 529–538. https://doi.org/10.3920/BM2015.0099
28. Wu, J., Xiong, Y., Zhong, M., Li, Y., Wan, H., Wu, D., Liu, Q., 2020. Effects of purified fibre-mixture supplementation of gestation diet on gut microbiota, immunity and reproductive performance of sows. J. Anim. Physiol. Anim. Nutr. 104, 1144–1154. https://doi.org/10.1111/jpn.13287
29. Wu, Y., Liu, W., Li, Q., Li, Y., Yan, Y., Huang, F., Wu, X., Zhou, Q., Shu, X., Ruan, Z., 2018. Dietary chlorogenic acid regulates gut microbiota, serum-free amino acids and colonic serotonin levels in growing pigs. Int. J. Food Sci. Nutr. 69, 566–573. https://doi.org/10.1080/09637486.2017.1394449

30. Xu, C.C., Yang, S.F., Zhu, L.H., Cai, X., Sheng, Y.S., Zhu, S.W., Xu, J.X., 2014. Regulation of N-acetyl cysteine on gut redox status and major microbiota in weaned piglets. J. Anim. Sci. 92, 1504–1511. https://doi.org/10.2527/jas.2013-6755
31. Yan, H., Lu, H., Almeida, V.V., Ward, M.G., Adeola, O., Nakatsu, C.H., Ajuwon, K.M., 2017. Effects of dietary resistant starch content on metabolic status, milk composition, and microbial profiling in lactating sows and on offspring performance. J. Anim. Physiol. Anim. Nutr. 101, 190–200. https://doi.org/10.1111/jpn.12440
32. Yan, H., Yu, B., Degroote, J., Spranghers, T., Van Noten, N., Majdeddin, M., Van Poucke, M., Peelman, L., De Vrieze, J., Boon, N., Gielen, I., Smet, S.D., Chen, D., Michiels, J., 2020. Antibiotic affects the gut microbiota composition and expression of genes related to lipid metabolism and myofiber types in skeletal muscle of piglets. BMC Vet. Res. 16, 392. https://doi.org/10.1186/s12917-020-02592-0
