# Supplementary material for: Effects of Major Families of Modulators on Performances and Gastrointestinal Microbiota of Poultry, Pigs and Ruminants: A Systematic Approach
Source: Microorganisms. 2023 May 31;11(6):1464. doi: 10.3390/microorganisms11061464 (PMC10300821; doi:10.3390/microorganisms11061464)
Supplement: Supplementary file 1 [file microorganisms-11-01464-s001.zip › Supplementary Data S3.pdf]

Supplemental Data S3: Effect of Modulators on Intestinal Microbiota and Phenotype of Ruminants

Table S31: Effect of antibiotics on the phenotype and gastro-intestinal microbiota of ruminants

Ref	Intervention	Conditions	Methods	Phenotype		Microbiota			
				Weight	Diarrhoea rate	Diversity	Phylum	Lactic acid-producing bacteria	SCFA-producing bacteria
Kim 2021	Neomycin	Calf<br>Male and Female<br>Diarrheic	Antibiotic<br>7 weeks<br>Sequencing	↑ BW	=	= β diversity	ND	Feces:<br>↑ Lactobacillus	Feces:<br>↓ Akkermansia<br>↑ Prevotella
Pereira 2018	Ceftiofur, Penicillin, Ampicillin and Oxytetracycline	Bos Taurus<br>Male<br>Healthy	Antibiotic (0.01µg/ml of ampicillin sodium, 0.1µg/ml of cefiofur sodium, 0.005µg/ml of penicillin G sodium, and 0.3 µg/ml of oxytetracycline hydrochloride)<br>5 weeks<br>Sequencing	ND	ND	= shanon index	= phylum abundance	ND	ND

Table S32: Effect of transplantation on the phenotype and gastro-intestinal microbiota of ruminants

Ref	Intervention	Conditions	Methods	Phenotype				Immunity	Microbiota			
				Weight	Food intake	FCR or FCE	Diarrhoea rate		Diversity	Phylum	Lactic acid-producing bacteria	SCFA-producing bacteria
Kim 2021	Fecal microbiota from healthy calves	Calf<br>Male and Female<br>Diarrheic	Transplantation (5g of feces)<br>7 weeks<br>Sequencing	↑ BW	ND	ND	↓	ND	Modification of the β-diversity	ND	Feces:<br>↑ Lactobacillus	Feces:<br>↓ Akkermansia<br>↑ Prevotella<br>↑ Bacteroides
Yin 2021	Rumen fluid from 3 months old sheep	Sheep<br>Female<br>Healthy	Transplantation (25mL of rumen fluid)<br>6 weeks<br>Sequencing	↓ ADG	↓ ADFI	=	ND	Serum:<br>= [Ig]<br>= [Antioxydant protein]<br>↑[Il-6]	= α-diversity<br>= β-diversity	Feces:<br>↓ Proteobacteria	ND	ND
	Rumen fluid from 1 year old sheep			↓ ADG	=	=	ND	Serum:<br>= [Antioxydant protein]<br>↑ [IgA]<br>↑ [Il-6]<br>↑ [INFα]	= α-diversity<br>Modification of β-diversity	Feces:<br>↓ Actinobacteria	ND	ND

Table S33: Effect of various interventions on the phenotype and gastro-intestinal microbiota of ruminants

Ref	Intervention	Conditions	Methods	Phenotype			Microbiota			
				Weight	Food intake	FCR or FCE	Diversity	Phylum	Lactic acid-producing bacteria	Pathogenic and/or Zoonotic bacteria
Hromadkova 2020	Colostrum	Holstein<br>Healthy	Colostrum (50% of milk)<br>72H<br>RTqPCR	ND	ND	ND	ND	ND	Ileum and Colon:<br>= Lactobacillus<br>= Bifidobacterium	Ileum and Colon:<br>= E.coli
			Colostrum (100%)<br>72H<br>RTqPCR	ND	ND	ND	ND	ND	Ileum and Colon:<br>↑ Lactobacillus<br>= Bifidobacterium	Colon:<br>↑ E.coli
Wickramasinghe 2020	Water	Holstein<br>Female<br>Healthy	Water available from day 0 (vs day 17)<br>10 weeks<br>Sequencing	ND	ND	ND	= shanon, simpson, ACE and Chao index<br>= β-diversity	= phylum abundance	ND	ND

Abbreviations:
BW: Body Weight
BWG: Body Weight Gain
ADG: Average Daily Gain
FCR: Feed Conversion Ratio
= No change compared with the control group
↑ Increase compared with the control group
↓ Decrease compared with the control group
ND: Not Determined

Bibliography:

1. Hromádková, J., Suzuki, Y., Pletts, S., Pyo, J., Ma, T., Chen, Y., Steele, M.A., Guan, L.L., 2020. Effect of colostrum feeding strategies on the expression of neuroendocrine genes and active gut mucosa-attached bacterial populations in neonatal calves. J. Dairy Sci. 103, 8629–8642. https://doi.org/10.3168/jds.2019-17710
2. Kim, H.S., Whon, T.W., Sung, H., Jeong, Y.-S., Jung, E.S., Shin, N.-R., Hyun, D.-W., Kim, P.S., Lee, J.-Y., Lee, C.H., Bae, J.-W., 2021. Longitudinal evaluation of fecal microbiota transplantation for ameliorating calf diarrhea and improving growth performance. Nat. Commun. 12, 161. https://doi.org/10.1038/s41467-020-20389-5
3. Pereira, R.V.V., Carroll, L.M., Lima, S., Foditsch, C., Siler, J.D., Bicalho, R.C., Warnick, L.D., 2018. Impacts of feeding preweaned calves milk containing drug residues on the functional profile of the fecal microbiota. Sci. Rep. 8, 554. https://doi.org/10.1038/s41598-017-19021-2
4. Wickramasinghe, H.K.J.P., Anast, J.M., Schmitz-Esser, S., Serão, N.V.L., Appuhamy, J. a. D.R.N., 2020. Beginning to offer drinking water at birth increases the species richness and the abundance of Faecalibacterium and Bifidobacterium in the gut of preweaned dairy calves. J. Dairy Sci. 103, 4262–4274. https://doi.org/10.3168/jds.2019-17258
5. Yin, X., Ji, S., Duan, C., Ju, S., Zhang, Y., Yan, H., Liu, Y., 2021. Rumen fluid transplantation affects growth performance of weaned lambs by altering gastrointestinal microbiota, immune function and feed digestibility. Anim. Int. J. Anim. Biosci. 15, 100076. https://doi.org/10.1016/j.animal.2020.100076
